# MultiNet: Improving Gaze Estimation via Face-to-Camera Distance and Multi-Branch Learning

**DOI:** 10.3390/s26196265

**Published:** 2026-10-02

**Authors:** Nikita Ligostaev, Nicola Conci, Roberto Passerone, Andrey Somov

**Affiliations:** 1 Center for Engineering Systems and Sciences, Moscow 121205, Russia; n.ligostaev@rcdei.com (N.L.); a.somov@centraluniversity.ru (A.S.); 2 Department of Information Engineering and Computer Science, University of Trento, 38123 Trento, Italy; nicola.conci@unitn.it; 3 Central University, Moscow 123056, Russia

**Keywords:** computer vision, deep learning, depth camera, eye tracking, gaze estimation, point of gaze, setup, visualization, web camera

## Abstract

We present MultiNet, a multi-branch pipeline that directly predicts the gaze vector or the point of gaze from multiple modalities derived from a single camera capture. In particular, we address the under-explored use of metric face-to-camera distance estimated from a conventional RGB web camera for appearance-based gaze estimation, in contrast to many distance-aware alternatives which require depth sensors, stereo rigs, or additional hardware. The proposed method employs a convolutional neural network (CNN) with attentional feature fusion and integrates cropped face images, left and right eye patches, head-pose information, and estimated camera-to-face distance. We also introduce NLGaze, a 141,000-image dataset collected for unconstrained and natural desktop settings, thereby emphasizing real-world applicability. In a person-specific real-time evaluation, MultiNet-Base reaches a root mean square error (RMSE) of 43.01 pixels at 15 frames per second (FPS) on a desktop with an off-the-shelf web-camera. We evaluate the proposed method against representative state-of-the-art solutions on benchmark datasets. MultiNet achieves angular errors of 2.94°, 7.47°, and 7.58° at best on the MPIIFaceGaze, Gaze360, and NLGaze datasets, respectively, demonstrating competitive or superior performance in appearance-based gaze estimation. The results of the ablation study show that the attentional feature fusion mechanism and the addition of the estimated distance input to the set of input modalities result in significant angular error reduction.

## 1. Introduction

Eye tracking, or oculography, measures ocular behavior and is a core sensing modality for visual human–computer interaction. It describes how the eyes move while a person observes visual stimuli [1]. A central problem in this area is *gaze estimation*, which infers where a person is looking within a screen or surrounding scene [2].

Gaze estimation has been studied through a broad spectrum of approaches, from heuristic procedures [3,4] to dedicated *eye-tracking* devices that measure small eye movements. Such devices differ in form factor and sensing technology. Most remote systems are non-invasive: they are placed near a display and monitor the eyes from a distance. A typical screen-based tracker combines a stimulus display with infrared cameras mounted below the screen so that corneal reflections can be detected [5]. The tracker then uses these reflections together with pupil position to estimate gaze on the screen or in the environment. Wearable trackers instead place cameras on glasses or similar devices to view the eyes from multiple angles. This study focuses on non-invasive screen-based solutions.

Infrared eye trackers can be highly accurate because infrared or near-infrared non-collimated illumination produces corneal reflections that are not visible in the ordinary RGB spectrum [6]. Nevertheless, screen-based trackers remain constrained in dynamic activities and everyday scenarios. They often assume that the participant keeps the head approximately fixed relative to the display [7]. Occlusions and artifacts caused by eyeglasses, reflections, or objects in the field of view can also interrupt tracking and reduce data quality [8,9]. For this reason, conventional eye trackers are especially effective in controlled laboratory studies but are less convenient for scalable deployment in natural desktop environments.

For less constrained use, researchers have proposed *appearance-based* gaze-estimation methods that operate with inexpensive RGB video devices, including web cameras [10,11,12]. These methods have achieved remarkable results with the advent of deep learning, demonstrating promising performance in within-domain evaluations [13,14]. However, their performance drops significantly when applied to real-time evaluation or deployed in environments that differ from the training setting. This is mostly due to the domain gap [15,16,17], because the model does not have access to any target domain data.

Distance information from the face to the camera can help to improve gaze estimation performance [18]. However, utilization of distance information, metric in particular, is still underinvestigated in 2D and/or 3D gaze estimation tasks (see Section 2). In this study, we propose a multi-branch method together with metric distance estimation for 2D and/or 3D gaze estimation in desktop scenarios. We propose direct mapping, interpreting the acquired images and inferring the observed points on the user screen or unit gaze vector originating from the middle point of the eyes. Our approach relies on a web camera to ensure a non-intrusive and scalable solution [19]. To address the shortcoming of appearance-based methods that use a single input image [20], we acquire multiple input modalities (cropped face, eyes, head pose, and estimated distance) together with the distance estimation metric as an additional input for the model. One of the novelties of this work is the demonstration of the practical feasibility of using metric distance information. Specifically, we show, using a commercial web-camera system, that distance information improves the robustness and accuracy of 2D and/or 3D gaze estimation in desktop scenarios.

The research gap targeted here is not depth sensing in general, but the practical use of metric distance inferred from a single RGB web camera as an explicit modality in appearance-based 2D and/or 3D gaze estimation. We additionally propose the NLGaze dataset, which was collected for natural desktop operation, with uncontrolled background and illumination conditions, rather than for laboratory-only calibration.

This study is a significant extension of our previous research [21]. In that work, we reported on real-time tracking with a deep learning model and provided comparisons with existing real-time solutions, such as GazeRecorder [22]. In contrast, in the current study. we develop an updated architecture that improves accuracy and present the processing methods and data statistics in greater depth. We highlight comparisons in offline tracking and search for the best configuration for appearance-based gaze estimation. We also compare the influence of the addition of artificial modalities and geometric features, such as distance from the face to the camera, with state-of-the-art methods that typically use a single full frame as input. An ablation study highlights the contribution of each input modality to the overall performance. Furthermore, a stability analysis using different types of noise is reported.

This article is organized as follows: in Section 2, we present the state-of-the-art and identify research gaps. In Section 3, we discuss the proposed methodology. Experimental results are presented in Section 4. The implications of the experimental findings are discussed in Section 5, and operational limitations and future research directions are summarized in Section 6. Concluding remarks are reported in Section 7.

## 2. Related Work

This section reviews the main directions in gaze estimation research, with emphasis on model-based methods, appearance-based methods, and distance-aware extensions.

### 2.1. Model-Based Gaze Estimation

Early model-based gaze-estimation pipelines represent the eye and surrounding face with simplified geometry rather than relying only on raw appearance. These representations are attractive because they provide an interpretable link between eye shape, head pose, and gaze direction.

Since the eyeball is approximately spherical and the pupil and iris are close to circular, many geometric approaches fit a generic 3D eye model to detected landmarks and infer gaze as the direction from the eyeball center toward the pupil center [23,24]. Their generalization is constrained by the diversity of available training data and the accuracy of 2D landmark localization under unseen conditions.

Later methods improved geometric fitting by using 2D facial landmarks as observations for 3D eye–face models, but real-time operation remained difficult. Wang et al. proposed a 3D deformable eye–face model to estimate gaze using observed 2D facial landmarks [25]. This approach, trained offline on data from multiple subjects, enabled the recovery of individual 3D eye–face models and personal eye parameters. However, such models may still encode factors unrelated to gaze, including person-specific appearance and image artifacts. Park et al. introduced a hybrid approach, combining geometric modeling with deep learning [26]. Their pipeline first converted the original image into an anatomical pictorial gaze representation with a CNN and then applied another CNN to estimate 3D gaze direction. This representation suppresses nuisance factors such as iris color, cosmetics, hair near the eyes, camera noise, blur, and compression artifacts, but its practical use is limited by the absence of real-time performance.

Overall, model-based methods depend heavily on accurate landmarks, which may require expensive annotation or fail under large head motion and occlusion. This limitation has motivated a shift toward appearance-based deep-learning methods, which can learn robustness to illumination, pose, and occlusion directly from image data.

### 2.2. Appearance-Based Gaze Estimation

Appearance-based gaze estimation methods have gained significant traction due to their ability to utilize image features directly as inputs for regression-based techniques [10,12,13,27], bypassing the need for manually defined geometric models. The most common approaches collect diverse and extensive gaze datasets [14,28] to train a model with robust generalization capabilities. This is very costly, and the diversity of the available data remains limited.

Subsequently, studies have attempted to reduce the amount of required training data while improving robustness to head movement. Adaptive linear regression increased tolerance to pose changes [29], whereas multiview camera systems enlarged the training distribution by synthesizing eye appearances from different viewpoints [30]. The MPIIGaze release further advanced the field by enabling standardized evaluation under diverse everyday conditions [13]. It introduced strong inter-personal and intra-personal variation and became a benchmark for both cross-person and person-specific gaze estimation.

Early appearance-based networks often processed a single eye crop, while later multi-region designs combined eye patches with face regions to improve accuracy [10,31]. Recent research has also investigated generative modeling, unsupervised learning, and attention mechanisms for more robust gaze inference [32,33,34,35]. Despite these advances, many methods still rely on high-resolution inputs, careful hyperparameter tuning, or computationally demanding components, which complicates real-time use.

Modern benchmarks and methods, including MPIIGaze [13], Gaze360 [28], and ETH-X-Gaze [14], report strong accuracy in controlled or well-characterized acquisition settings but still degrade under more open real-world conditions. Typical errors can remain near 2–5 degrees in laboratory scenarios [5,12,14], increase on laptop data [20], and become substantially larger outdoors [28]. Furthermore, converting a 3D gaze vector into a usable 2D screen point usually requires calibration and knowledge of display geometry, reducing direct deployability.

To circumvent these geometric complexities, an alternative approach is to directly estimate the 2D on-screen gaze point. Methods leveraging datasets like GazeCapture [10] and MPIIFaceGaze [20] frame this predominantly as a regression task, predicting the continuous (x,y) coordinates on the screen. Other works have explored casting the estimation as a classification problem; for instance, Luo et al. [36] discretized the screen into 21 fixed areas and employed an improved Xception network to classify the gaze region from binocular images. Another study, such as that of Ansari et al. [37], focused on developing a 2D gaze estimation CNN on low-quality web-camera images. Using training of a person-specific model, this research showed that a model created for one person yields much lower errors than general models trained on multiple users’ data, which fall short in generalizing to new users and different domains. Despite the direct output of 2D or 3D gaze estimation methods, they face their own set of challenges. High computational demands, long-term gaze stability, latency, and the sensitivity of models to variations in lighting, occlusions, and head movements remain unresolved.

### 2.3. Distance for Gaze Estimation

In desktop environments, appearance-based methods typically focus on a single input that is further processed by the model, trying to extract the maximum valuable information [38]. Conversely, for tablets, mobiles, or edge devices, appearance-based methods rely on *multi-branch* networks [10,39]. For example, the seminal work by Krafka et al. introduced iTracker, the first large-scale gaze estimation dataset for mobile and tablet devices, using a CNN with cropped face, eyes, and head pose as input without calibration [10]. Recent research on gaze estimation for mobiles follows a similar multi-branch logic, adding a calibration procedure. The study by Park et al. [39] is an improvement on the research discussed earlier. The inputs of their model are left/right eye images, left/right eye sizes, Euler angles x,y,z, and the center point of the face. Unlike the iTracker model, the authors do not adopt face images as input and use grayscale images instead of RGB.

The proposed MultiNet differs from these earlier multi-branch architectures in three technical aspects. First, it is designed for desktop RGB web-camera operation and processes cropped face, left eye, right eye, head pose, and metric distance modalities derived from the same frame, whereas iTracker was primarily designed for mobile and tablet devices and does not use metric face-to-camera distance. Second, the full MultiNet model uses separate ResNet50-based encoders for all modalities, including a distance-map encoder, while MultiNet-Base uses lightweight task-specific encoders for real-time desktop inference. Third, MultiNet does not simply concatenate branch outputs: it integrates eye, face, head pose, and distance features through pair-sequential attentional feature fusion blocks, allowing modality interactions to be weighted before regression.

The appearance-based methods presented in the literature neglect distance information, which means they are not distance-aware. These methods can help understand the environment to some extent by learning from data. To the best of our knowledge, adding the metric distance as an additional input for gaze estimation on low-cost devices, such as web cameras, has not been thoroughly discussed in the field. Nevertheless, using metric distance is not a completely new idea and was partially adopted by Lian et al., who relied on the use of a depth camera (RGBD) [18]. The authors used depth maps to decompose the gaze point estimation into eyeball pose, head pose, and 3D eye position estimation. They further designed a CNN-based network to simultaneously refine depth maps and predict the gaze point.

Such depth-aware pipelines demonstrate the usefulness of geometry, but they also depend on specialized hardware, including stereo cameras, cameras that combine stereo with active near-infrared (NIR) illumination, and RGBD (depth) cameras that combine image and time-of-flight (ToF) sensors, or sensor-specific calibration that is often unavailable on ordinary desktop computers [18,40]. While useful for various applications in robotics and human–computer interaction (HCI) research more broadly, such hardware limits the widespread adoption of dedicated distance- and depth-aware methods. Methods that can instead imitate RGBD cameras using a single low-cost RGB camera therefore carry clear practical value. The most recent appearance-based gaze estimation methods that show inference results also do not utilize distance explicitly [41,42].

In conclusion, although considerable progress has been made in the field of appearance-based gaze estimation, significant shortcomings remain. The current methods are not yet suitable for immediate deployment in real-time systems due to unstable predictions in different settings, mostly caused by previously unseen setups and illumination, particularly in dynamic and unconstrained environments. Furthermore, the idea of using a metric distance with a low-cost device requires further research.

In this study, we explore the use of a multi-branch network with a distance estimation block from a web camera as conditioning to improve gaze estimation performance in comparison to appearance-based methods that typically use a single image as input. The proposed formulation therefore preserves the practical advantages of RGB-only acquisition while injecting a metric cue that is usually available only from specialized depth hardware.

## 3. Methods

In this section, we present in detail our proposed solution, MultiNet. First, we provide the reasoning behind and the motivation for data collection that were not described in our previous work [21]. Next, we describe the architecture of the proposed method and present the training procedure for 2D and/or 3D gaze estimation.

### 3.1. Data Collection

The use of proprietary datasets for gaze estimation offers several advantages in scenarios where access to 2D points of gaze (PoGs) and the distance between the user’s face and the screen is limited. Each dataset is tailored to a specific setup, allowing the environment to be adapted to specific needs. It should be noted that there are a decent number of large datasets in this area, but most of them are collected in a controlled environment, which makes them difficult to use in normal, uncontrolled scenarios (refer to Table 1 for additional details).

The most common datasets currently available in the literature are MPIIFaceGaze [20], ETH-XGaze [14], GazeCapture [10], and EVE [5], presented in Table 1. MPIIFaceGaze, an extension of MPIIGaze [13], lacks full frames extracted from the camera, which limits the diversity of gaze sources and introduces uncertainties that may not be generalized by the model. ETH-XGaze was collected in a controlled environment with chromakey, making it unsuitable for real-world, in-the-wild gaze estimation, which is the focus of this study. GazeCapture includes data from a variety of mobile devices with camera resolutions ranging from 640 × 480 to 1280 × 720 pixels, which poses challenges for gaze estimation, particularly in desktop environments where consistent device specifications are critical. Also, EVE was collected in a controlled environment with chromakey, further limiting its applicability to in-the-wild scenarios.

In our solution, we do not use controlled lighting sources, such as light boxes or a fixed background, e.g., chromakey. We propose to develop a complete pipeline, including data collection to ensure adaptation to challenging environments and in-the-wild adoption.

We collect our dataset by plotting a *moving* reference PoG, which the user is required to look at, and capturing frames that contain other useful information at defined moments in time. The process works as follows. First, a *gaze map* is defined: the gaze map is a coarsely discretized representation of the screen space to more easily ensure full coverage of the screen area during data collection. For that, we use a scaling factor μ=30, which corresponds to partitioning the screen in 1920/μ=64 columns and 1080/μ=36 rows of rectangular 30×30-pixel areas, for a total of 36×64=2304 regions. Then, the user is asked to continuously follow a moving dot on the screen. The moving dot is displayed on the screen according to the following criteria:

1. The initial position of the moving point is randomly defined within the screen region.
2. During each iteration, the coordinates are updated and captured in the gaze map to keep track of how many times a specific region has been tested; this provides a density of screen coverage, defined as the number of fixations occurring at a specific rectangle area of the gaze map.
3. The dot does not simply move discontinuously from the initial to the final point. Instead, it moves along a straight line to the new target coordinates. During every iteration, the target coordinate is identified as a random point within the minimal density area of the gaze map.
4. During the trajectory, a random perturbation sampled from a normal distribution is added to the current x,y coordinates of the moving point to ensure that the user is unable to predict the subsequent position of the point. This approach results in a non-linear path.

The gaze vector in the camera coordinate system, used for the 3D gaze estimation task, can be derived from the recorded point of gaze (PoG) using the following information: the camera intrinsics, calibrated with the OpenCV framework and a checkerboard of known size, and the extrinsic camera transformation, determined using a first-surface mirror [45].

### 3.2. Dataset Description

For data collection, we use an RGB camera, with the estimated distance from the face to the screen serving as a key parameter. The distance is estimated from the full frame, using two-dimensional landmarks extracted via MediaPipe (MediaPipe face landmark detector, https://ai.google.dev/edge/mediapipe/solutions/guide, “URL (accessed on 6 August 2026)”) [46] and a generic three-dimensional face mesh model. This is accomplished through the resolution of a perspective-n-point (PnP) problem. Given the camera parameters after camera calibration, represented by the intrinsic camera matrix K and the extrinsic matrix $\left[\begin{array}{c} R|t \end{array}\right]$, as well as the canonical face mesh $F\in R^{3\times 468}$ from MediaPipe, we compute the estimated distance, as shown in Algorithm 1.
**Algorithm 1** PnP-based Face-to-Camera Distance Estimation**Input:** full frame (RGB) sample: $x_{i}\in R^{3\times H\times W}$;            intrinsic camera matrix: $K\in R^{3\times 3}$;            specific points (eye, mouth corners, nose) in face geometry model: $G\in R^{7\times 3}$;            face geometry model: $F\in R^{468\times 3}$;            landmark estimator: *l*;            landmark IDs: $L\in R^{7}$;            number of iterations for numerical stability: $K_{in}$**Output:** estimated distance $\hat{d}$Extract all 2D face landmarks: $f_{i}\leftarrow l\left(x_{i}\right)$;Extract specific 2D face landmarks: $f_{i}\leftarrow f_{i}\left[L\right]$;Utilize PnP Ransac:$R_{i},t_{i}=\mathrm{None},\mathrm{None}$;$R_{i},t_{i}\leftarrow \mathrm{cv}2.\mathrm{solvePnPRansac}\left(G,f_{i},K,R_{i},t_{i}\right)$;**for** $n=1,2,\ldots ,K_{in}$ **do**      Utilize iterative PnP:      $R_{i},t_{i}\leftarrow \mathrm{cv}2.\mathrm{solvePnP}\left(G,f_{i},K,R_{i},t_{i}\right)$;**end for**Get rotation matrix: $R\leftarrow \mathrm{cv}2.\mathrm{Rodrigues}(R_{i})$;Transform full face model: $F_{\mathrm{all}}\leftarrow R\cdot F^{T}+t_{i}$;Calculate distance from face to camera: ${\hat{d}}_{i}\leftarrow {F_{\mathrm{all}}}_{9,2}$;**Return:** ${\hat{d}}_{i}$

**Figure 1:**
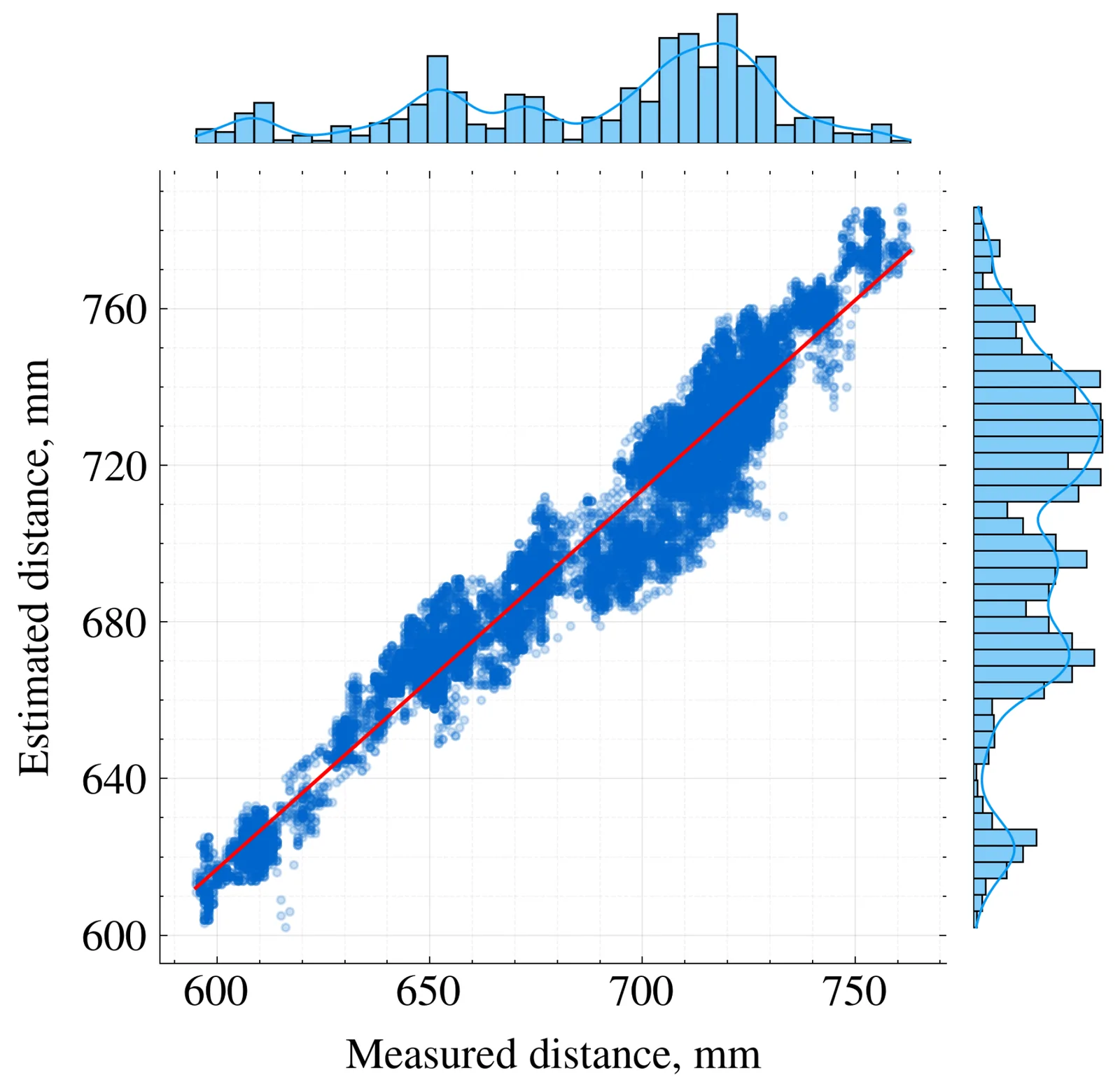
Estimated and measured distance correlation in NLGaze.

To validate this approach and as a confirmation of our setup, we have employed a depth camera to ascertain the correlation between the estimated and measured distances. This provides empirical evidence to support the rationale behind the estimation method (see Figure 1). More precisely, the coefficient of determination is $R^{2}=0.81$, the mean absolute percentage error is MAPE=2.2%, the Spearman’s rank correlation coefficient $r_{s}=0.96$, and the Pearson’s correlation coefficient $r_{p}=0.97$, which show that our predictive model captures more than 80% of the data variance, and the two variables have a strong linear and monotonic relationship as expected.

The proposed dataset, designated as NLGaze, contains 21 subjects (16 males and 5 females) aged between 19 and 26 years. It comprises approximately 141,000 RGB images collected in naturalistic desktop conditions, specifically office rooms equipped with window shades and ample natural light streaming in from outside. Unlike datasets acquired with fixed illumination, chromakey backgrounds, or a strictly constrained head pose, NLGaze was recorded with ordinary indoor backgrounds and mixed natural and artificial illumination. The collected dataset has a natural bias towards working hours, not covering challenging illumination presented with over- or under-exposed samples. The illumination levels were not controlled explicitly and were not measured. To provide a more challenging environment for the extraction of relevant gaze features, both the training and test sets include subjects presented with and without glasses; 16 subjects are without visual aids. The data in the proposed dataset are anonymized.

### 3.3. Model Input

The aim of MultiNet is to learn a model that can estimate a 2D point of gaze or a unit gaze vector from a processed camera frame that gives us multiple images. To do so, we decompose the task into two phases, namely the preprocessing of input data and the gaze estimation. The input consists of four RGB images corresponding to the cropped face $x_{F}\in R^{3\times H\times W}$, the right eye $x_{R}\in R^{3\times H\times W}$, the left eye $x_{L}\in R^{3\times H\times W}$, and the head pose $x_{H}\in R^{3\times H\times W}$, plus the estimated distance $\hat{d}\in R$. The output is the predicted two-dimensional point of gaze $\hat{p}\in R^{2}$ or two-dimensional gaze vector $\hat{g}\in R^{2}$ in pitch and yaw depending on the gaze estimation task.

#### 3.3.1. Classical Image Normalization

**Figure 2:**
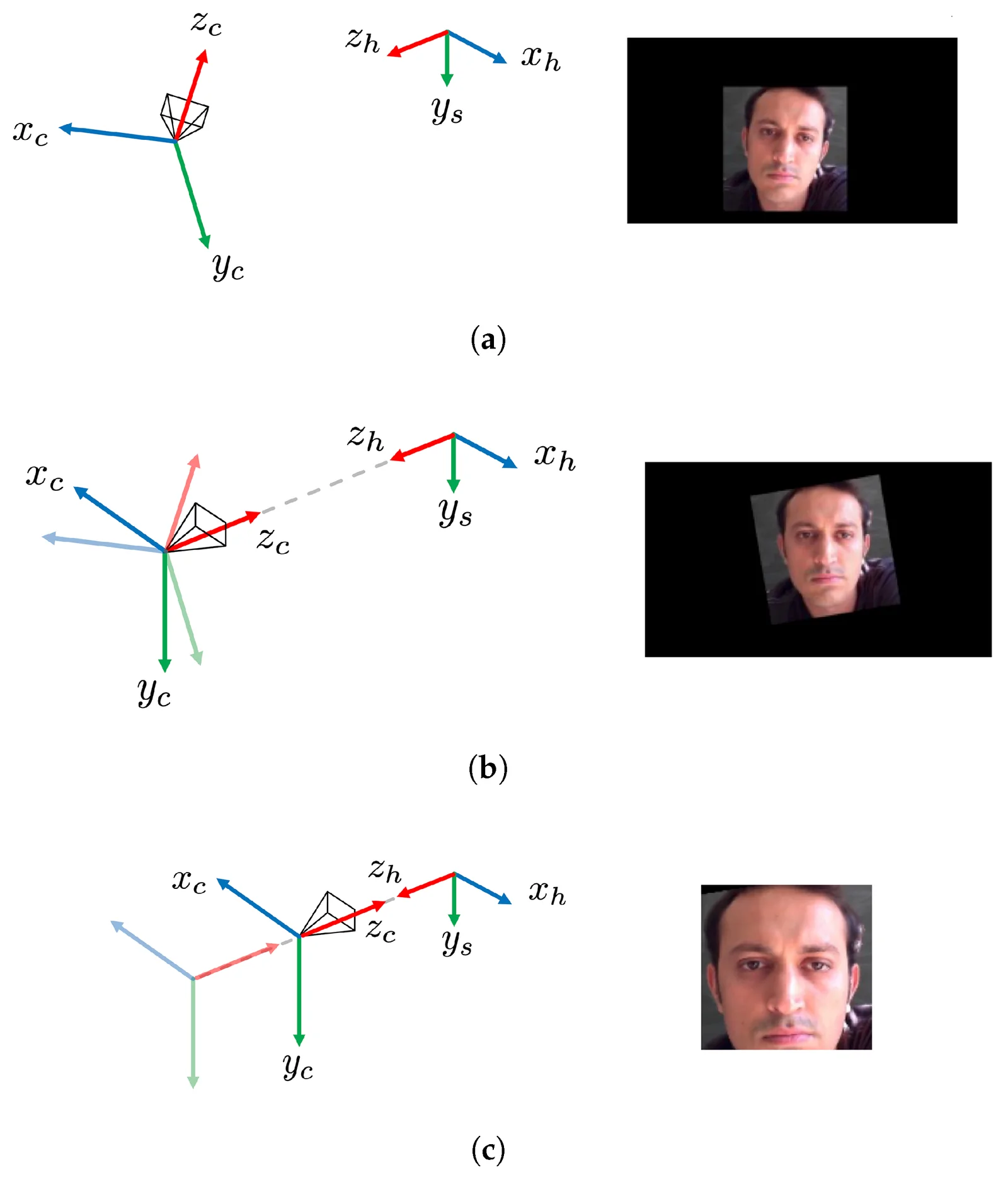
Image normalization proposed by Sugano et al. [30]. We visualize transformations with a virtual camera and provide visual examples using the sample from MPIIFaceGaze [20] (best viewed in color.) (**a**) Initial relationship between the head pose coordinate system *h*, centered at gaze origin $g_{o}$, and the camera coordinate system; (**b**) rotation of the camera coordinate system by $R_{c}$; (**c**) the normalized image after applying transformation matrix W that is equivalent to the one captured with a normalized camera.

When dealing with images in this research area, it is common practice to normalize the image size, ensuring a fixed size of the data to be processed when the distance of the user from the camera changes. Sugano et al. [30] introduced the concept of image normalization as a means of limiting the range of head pose variations when synthesizing training data for learning-based gaze estimation from 3D face meshes. The underlying concept is as follows: the virtual camera is rotated such that the camera coordinate system, the head pose coordinate system, and the world coordinate system are all centered, and the latter is scaled to place the virtual camera at a fixed distance from the image (Figure 2).

Formally, this procedure is defined as a perspective image warping with the transformation matrix $W=C_{n}S_{c}R_{c}K^{-1}$ (Figure 2a–c), where K is the original camera projection matrix obtained from the camera calibration, $S_{c}$ is a scaling matrix, $R_{c}$ is a rotation matrix, and $C_{n}$ is the projection matrix of the virtual camera, defined as follows:

(1) $$C_{n}=\left[\begin{array}{ccc} {\hat{f}}_{x} & 0 & {\hat{c}}_{x}/2\\ 0 & {\hat{f}}_{y} & {\hat{c}}_{y}/2\\ 0 & 0 & 1 \end{array}\right],$$

where ${\hat{c}}_{x},{\hat{c}}_{y}$ is the principal point of the virtual camera, i.e., the center of the normalized image, and ${\hat{f}}_{x},{\hat{f}}_{y}$ are the focal lengths in the *x* and *y* axes of the virtual camera. The same procedure can be used to extract normalized eye patches by defining the gaze origin $g_{o}$ as the center of a given iris (left or right) from the landmark estimator.

#### 3.3.2. Proposed Image Normalization

The proposed approach differs from the classical technique introduced by Sugano et al. [30], which involves the computation of virtual camera rotations and scaling based on precise camera calibration and three-dimensional transformations. In contrast, we utilize the direct affine transformations derived from the detected facial landmarks through MediaPipe [46]. This method is computationally efficient, robust to diverse experimental setup facilities, and suitable for real-time applications. In contrast to the approach proposed in [30], which relies exclusively on aligned images, our methodology encompasses the evaluation of both the aligned and non-aligned cropped versions of the face. Notations that are used in the proposed image normalization are provided in Table 2.

**Figure 3:**
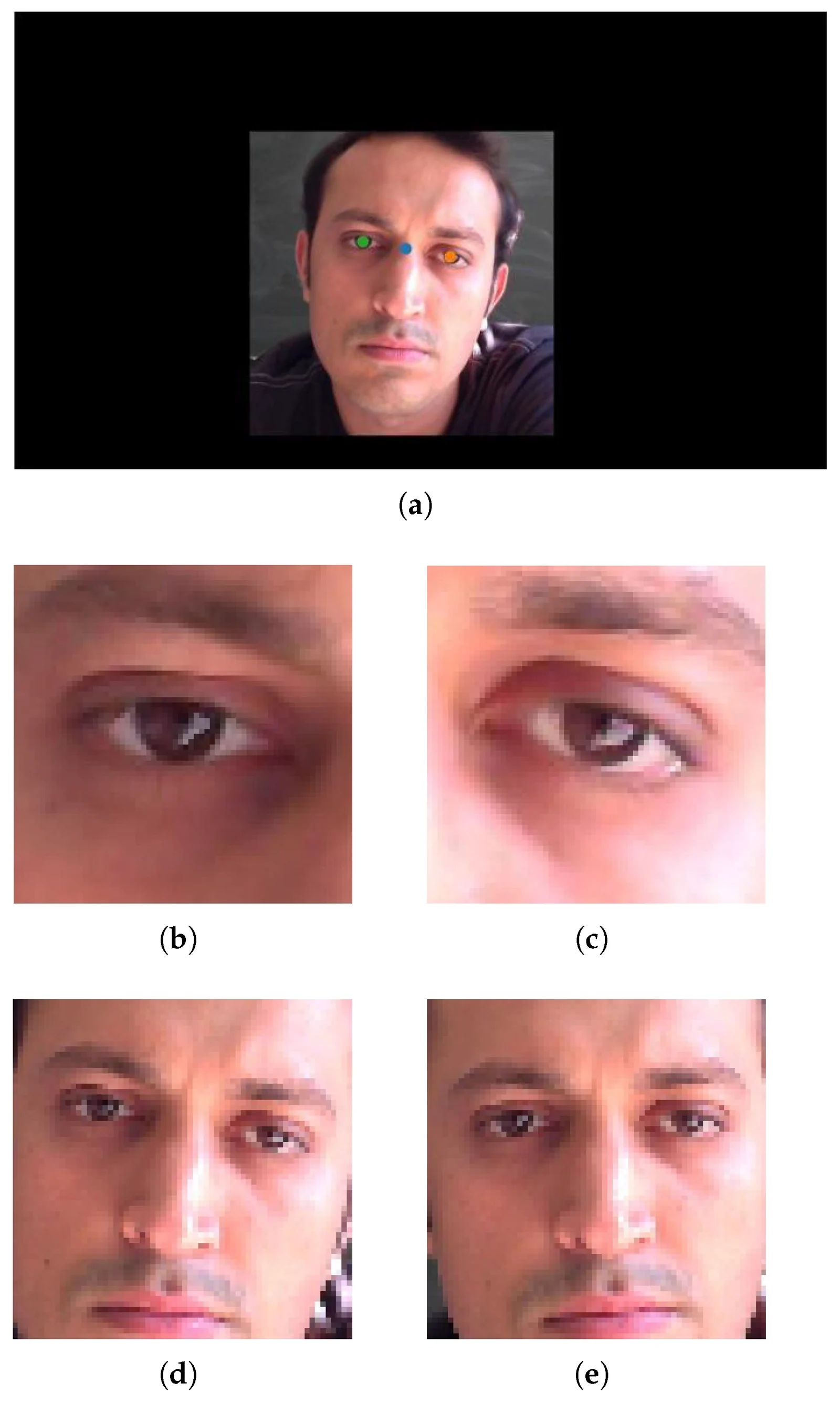
Proposed image normalization. We visualize transformations and provide visual examples using the sample from MPIIFaceGaze [20] (best viewed in color.) (**a**) Acquired RGB image; the colored dots are the coordinates of the eyes that are obtained by the landmark estimator extraction: green—right eye center, orange—left eye center, and blue—center between left and right eyes. (**b**) Normalized right eye patch; (**c**) normalized left eye patch; (**d**) normalized face patch without alignment; (**e**) normalized face patch with alignment.

**Cropped Left and Right Eye Images.** After face detection with the MediaPipe landmark estimator, the extracted landmarks are used to obtain the patches. The scaling factor for image transformations is defined as $s=d_{orig}/d_{n}$. The angle of head rotation, denoted by the variable θ, is used for the purpose of alignment. This angle is approximated based on the centers of the eyes, which are determined by the landmark estimator (Figure 3a). The angle is calculated as follows:

(2) $$\theta =\arctan\left(\frac{dy}{dx}\right),$$

where $dy=y_{r}-y_{l}$ and $dx=x_{r}-x_{l}$.

Conversely, it is not necessary to align the eye images, so in this case, the angle of rotation is set to zero degrees (θ=0°).

The transformation matrix M is defined as follows:

(3) $$\begin{array}{cc} M & =\left[\begin{array}{ccc} \alpha & \beta & \left(1-\alpha \right)\cdot c_{x}-\beta \cdot c_{y}\\ -\beta & \alpha & \beta \cdot c_{x}+\left(1-\alpha \right)\cdot c_{y} \end{array}\right]\\ \; & =\left[\begin{array}{ccc} s & 0 & \left(1-s\right)\cdot c_{x}\\ 0 & s & \left(1-s\right)\cdot c_{y} \end{array}\right], \end{array}$$

where α=s·cos(θ), β=s·sin(θ), and $\left(c_{x},c_{y}\right)$ are the coordinates of the center of the image, and $\left(x_{l},y_{l}\right)$ and $\left(x_{r},y_{r}\right)$ are the left and right eye centers, respectively.

Subsequently, the translation offset $t_{x},t_{y}$ is incorporated into the transformation matrix M to obtain an affine transformation W that is applied to the frame (Figure 3a):

(4) $$\begin{array}{c} W=\left[\begin{array}{ccc} s & 0 & \left(1-s\right)\cdot c_{x}+t_{x}\\ 0 & s & \left(1-s\right)\cdot c_{y}+t_{y} \end{array}\right], \end{array}$$

where $t_{x},t_{y}$ is the translation offset for left or right eye. The translation offset for the left eye is defined as follows:

(5) $$\begin{array}{c} t_{x}=0.5\cdot h-x_{l},\\ t_{y}=0.5\cdot h-y_{l}. \end{array}$$

Similarly, for the right eye, $t_{x}=0.5\cdot h-x_{r}$ and $t_{y}=0.5\cdot h-y_{r}$. This is carried out to ensure that the eyes are always centered in the cropped frame (Figure 3b,c).

**Cropped Face and Aligned Cropped Face Images.** The same approach as in the eye images ‘normalization’ is then employed to generate both the aligned and the misaligned versions of the cropped face image. The scaling factor is defined as the ratio of the desired distance to the eye centers, expressed as $s=d_{des}/d_{eyes}$.

As previously described, the translation offset is added to the transformation matrix M (3). The values of this offset are calculated as follows:

(6) $$\begin{array}{c} t_{x}=0.5\cdot h-\left(x_{l}+x_{r}\right)/2,\\ t_{y}=0.35\cdot h-\left(y_{l}+y_{r}\right)/2. \end{array}$$

The desired distance, $d_{des}$, and translation vectors, $t_{x}$ and $t_{y}$, are defined in a manner that maintains the eyes and face at specific positions in the frame (eyes are positioned on the line shifted by 35% from the top of the cropped frame, and the face is centered within the frame).

The final step is to apply the affine transformation matrix W (7) to obtain both the aligned (2) and non-aligned (θ=0°) versions of the image (Figure 3d,e):

(7) $$\begin{array}{c} W=\left[\begin{array}{ccc} \alpha & \beta & \left(1-\alpha \right)\cdot c_{x}-\beta \cdot c_{y}+t_{x}\\ -\beta & \alpha & \beta \cdot c_{x}+\left(1-\alpha \right)\cdot c_{y}+t_{y} \end{array}\right], \end{array}$$

where α=s·cos(θ), β=s·sin(θ), and $\left(c_{x},c_{y}\right)$ is the coordinate of the center of the image (left $\left(x_{l},y_{l}\right)$ and right $\left(x_{r},y_{r}\right)$ eye centers). Translation offsets $t_{x}$ and $t_{y}$ are defined in (7).

**Head Pose Images.** The head pose image is defined by cv2.boundingRect from the OpenCV (OpenCV Python library, https://docs.opencv.org/4.x/index.html, tested on Python 3.9.23, “URL (accessed on 6 August 2026)”) library from  the identified facial landmarks. Then, it is reshaped to make the image sizes consistent: $x_{H}\in R^{3\times H\times W}$.

### 3.4. Gaze Estimation

**Figure 4:**
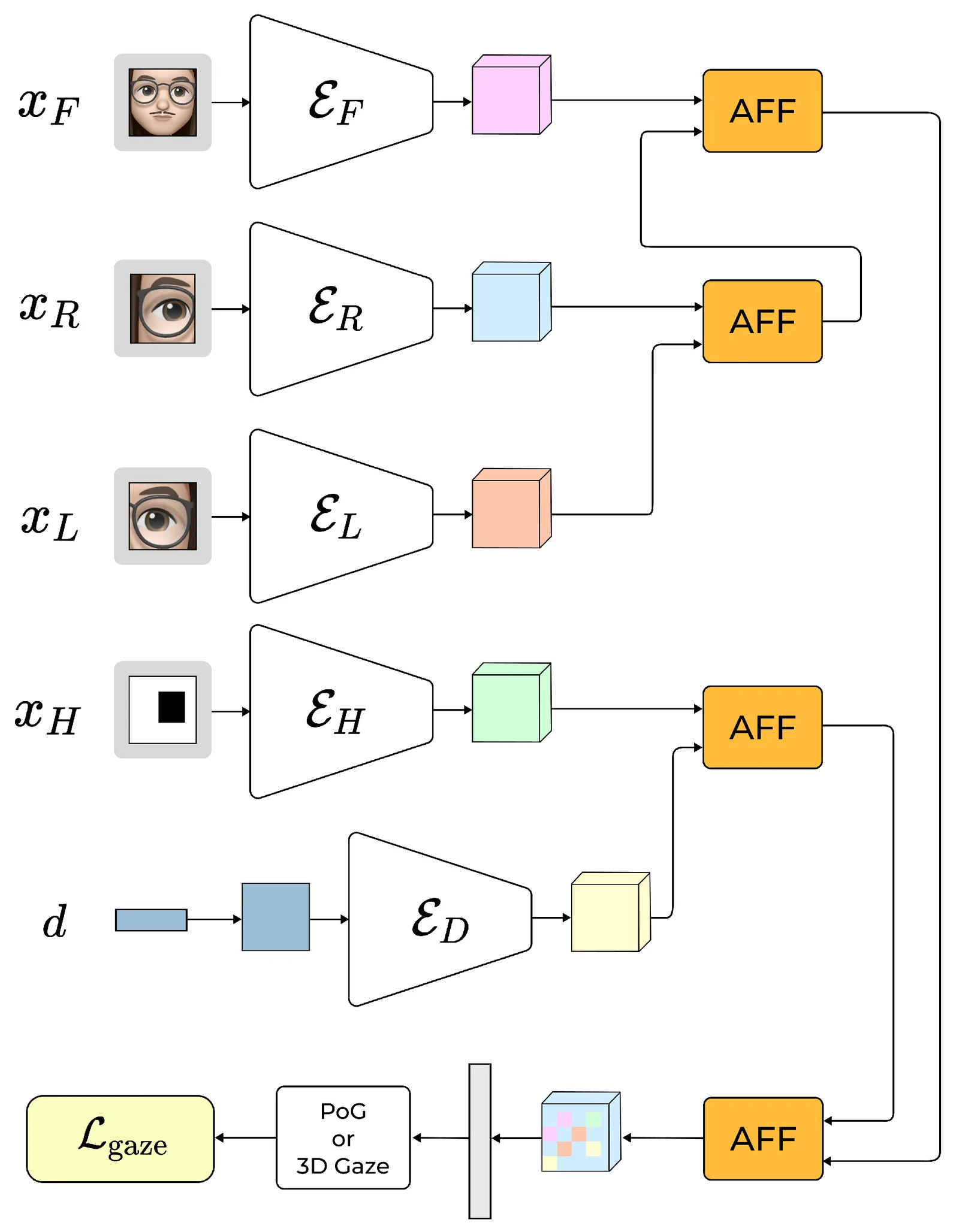
Overview of the proposed MultiNet architecture.

As shown in Figure 4, the MultiNet model takes four images as input, namely $x_{F}$, $x_{L}$, $x_{R}$, $x_{H}$, and the estimated distance *d* that is mapped to the distance map $d\mapsto x_{D}\in R^{3\times H\times W}$ as the input. The size of the input RGB images is set to 224×224. Higher resolution images would result in better gaze estimation performance [47]; however, this leads to higher computational load, forcing us to find a trade-off to ensure reasonable outcomes at an acceptable cost. The images are fed separately to vision encoders, which perform feature extraction. The outputs of the five encoders are passed pairwise through attentional feature fusion blocks [48], resulting in a unified feature map. This map is passed through a fully connected layer for solving a regression task (prediction of PoG or gaze direction).

In simple terms, our gaze estimation network can be divided into two subnets: a vision encoder for feature extraction and a fully connected (FC) layer to solve the regression task for gaze estimation. $L_{gaze}$ is utilized to optimize the gaze estimation network:

(8) $$L_{gaze}={∥g-\hat{g}∥}_{1},$$

where $\hat{g}$ and *g* are the predicted and corresponding actual gaze directions.

In MultiNet, we extract features using five backbones. We choose to convert d∈R to a pseudo depth map the same size as the input images ($x_{F}$, $x_{L}$, $x_{R}$, and $x_{H}$): $d\mapsto x_{D}\in R^{3\times H\times W}$. Each modality $x_{i}$ is the input to its own vision encoder $E_{i}$ (the *i* subscript in this subsection refers to the modality type, thus i∈{F,L,R,H,D}). As a result, we have a distance map vision encoder $E_{D}$, a head pose vision encoder $E_{H}$, left and right eye vision encoders $E_{L},E_{R}$, and a face vision encoder $E_{F}$ (Figure 4).

We first encode each image into an *x*-channel feature map as follows:

(9) $$c_{i}=E_{i}\left(x_{i}\right),$$

where $E_{i}$ is a ResNet50 feature extractor pretrained on ImageNet-1K. Simultaneously, we define decoders for each extracted feature map for image reconstruction purposes:

(10) $${\hat{x}}_{i}=D_{i}\left(c_{i}\right),$$

where $D_{i}$ is a lightweight CNN-based decoder, and ${\hat{x}}_{i}$ is the image reconstructed by the decoder. Each decoder $D_{i}$ is based on convolutions that progressively upsample the features from 256 channels to a 3-channel 224×224 RGB output (the same size as the input) using transpose convolutions, bilinear upsampling, and GroupNorm.

After processing the input images through the vision encoders (ResNet50 blocks), we have five separate 2048-channel feature maps. To integrate these maps into a unified representation, we employ an attentional feature fusion (AFF) block based on the multi scale channel attention module (MS-CAM) introduced in [48].

The AFF block operates as follows. Take for instance the first pair of feature maps from the vision encoders (e.g., the eyes): $C_{L}=E_{L}\left(x_{L}\right)$ and $C_{R}=E_{R}\left(x_{R}\right)$. The fusion process comprises two main steps: the self-attention step and the attentional weight summation step. In the self-attention step, channel-level local and global attention mechanisms are applied to the feature maps:

(11) $$\begin{array}{cc} A_{C_{L}} & =C_{L}⊙M\left(C_{L}\right),\\ A_{C_{R}} & =C_{R}⊙M\left(C_{R}\right), \end{array}$$

where $A_{C_{L}}$ and $A_{C_{L}}$ are attention-enhanced features, M is the multi scale channel attention module, and ⊙ is element-wise multiplication. This step is followed by feature integration, which consists of the normalization of the sum of the attention-enhanced features from both modalities:

(12) $$A_{\mathrm{int}}=\frac{A_{C_{L}}}{M\left(A_{C_{L}}\right)+M\left(A_{C_{R}}\right)}+\frac{A_{C_{R}}}{M\left(A_{C_{L}}\right)+M\left(A_{C_{R}}\right)},$$

where $A_{\mathrm{int}}$ is the integrated feature. Finally, the attentional feature fusion is defined as follows:

(13) $$\begin{array}{cc} F_{\mathrm{eyes}} & =F_{\mathrm{fusion}}\left(C_{L},C_{R}\right)\\ & =A_{c_{L}}⊙M\left(A_{\mathrm{int}}\right)+A_{c_{R}}⊙\left(1-M\left(A_{\mathrm{int}}\right)\right), \end{array}$$

where $F_{\mathrm{fusion}}$ is the fusion operation of the AFF block.

This process is applied in a pair-sequential fashion to fuse all available information into an integrated representation *z*, as outlined by the following equations:

(14) $$\begin{array}{cc} C_{F}=E_{F}\left(x_{F}\right);\;C_{L} & =E_{L}\left(x_{L}\right)\;\mathrm{and}\;C_{R}=E_{R}\left(x_{R}\right),\\ C_{H}=E_{H}\left(x_{H}\right); & \;C_{D}=E_{D}\left(x_{D}\right), \end{array}$$

(15) $$\begin{array}{cc} F_{\mathrm{eyes}} & =F_{\mathrm{fusion}}\left(C_{L},C_{R}\right),\\ F_{\mathrm{face},\;\mathrm{eyes}} & =F_{\mathrm{fusion}}\left(C_{F},F_{\mathrm{eyes}}\right),\\ F_{\mathrm{dist},\;\mathrm{hp}} & =F_{\mathrm{fusion}}\left(C_{D},C_{H}\right), \end{array}$$

(16) $$\begin{array}{cc} z & =F_{\mathrm{fusion}}\left(F_{\mathrm{face},\;\mathrm{eyes}},F_{\mathrm{dist},\;\mathrm{hp}}\right). \end{array}$$

Finally, *z* features are passed through a fully connected layer, which maps them to two regression targets, representing the model predictions. The training details of the network are described in the following subsection.

**Figure 5:**
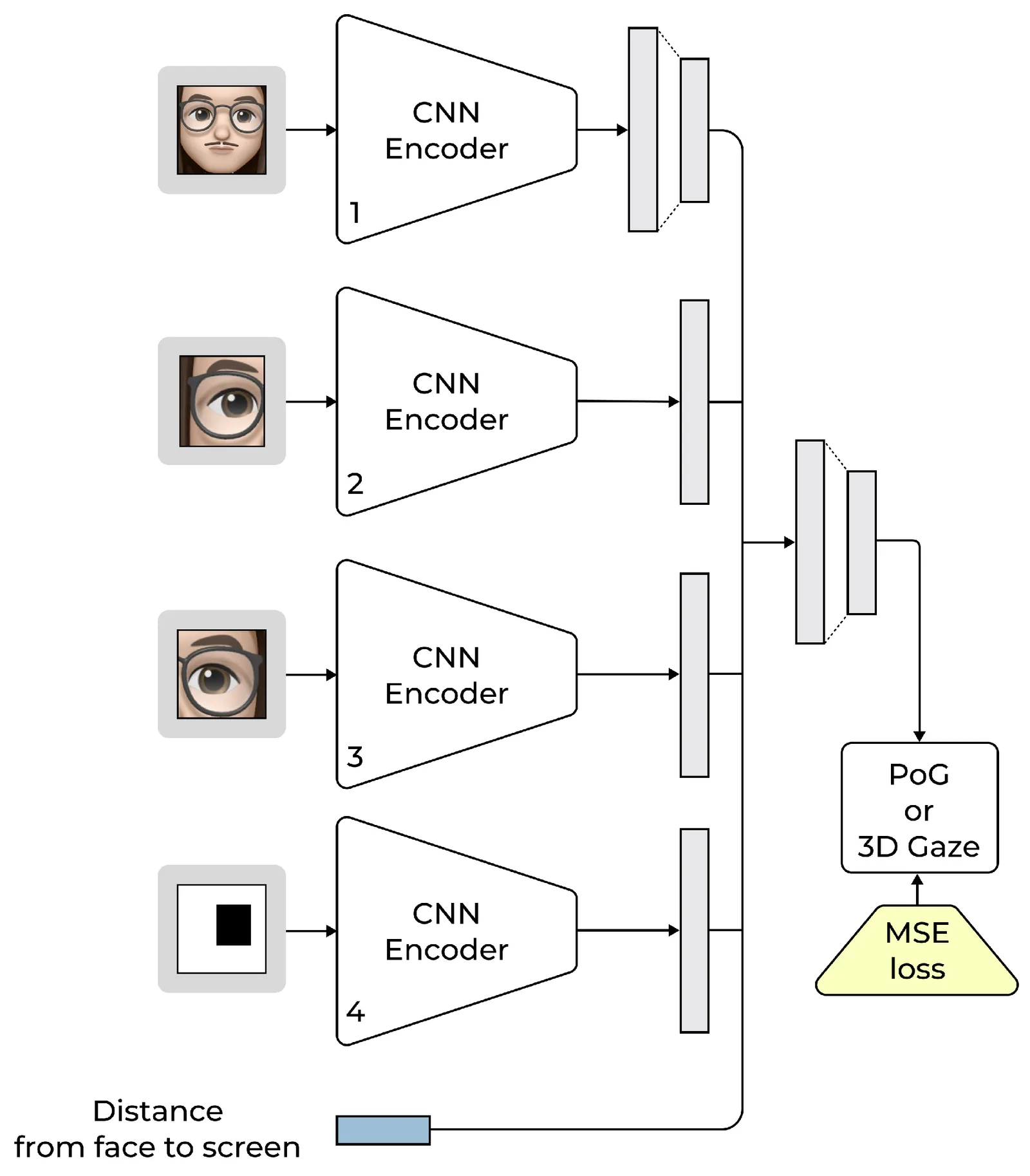
Overview of the MultiNet-Base architecture.

In addition to the full model, we have devised a lightweight network that can be easily used for real-time inference. We call it MultiNet-Base (Figure 5). This network configuration follows the main idea behind a multi-branch convolutional network, while reducing some blocks and using lightweight analogs. In MultiNet-Base, we use custom configurable CNN-based vision encoders instead of ResNet50, concatenation of flattened feature vectors instead of attentional feature fusion, and MLP with ReLU activations and dropout layers for PoG/gaze direction regression.

In our implementation, we made sure to adjust the hyperparameters of the model, with further tuning with a tuning framework (Ray Tune hyperparameter tuning framework, https://docs.ray.io/en/latest/tune/index.html, “URL (accessed on 6 August 2026)”), after which we obtain the best configuration of the network parameters. Notice that the face encoder processes the global facial features, while the left and right eye encoders capture fine-grained details, and the head pose encoder provides the spatial orientation information. Each of these encoders shares a common structural design but is parameterized separately to account for the varying complexities of the inputs. We detail the best configuration achieved after tuning next.

The **face encoder** starts with a convolutional layer input, which uses a 7×7 kernel with 3 input and 64 output channels. This is followed by two convolutional blocks. The first block consists of a 7×7 convolutional layer with 64 input and 128 output channels, followed by a ReLU activation function, batch normalization, and a 2×2 max-pooling layer. The second block follows a similar structure, with a 7×7 convolutional layer increasing the feature depth to 256, followed by ReLU activation, batch normalization, and max pooling.

Each **left eye encoder** and **right eye encoder** begins with a convolutional layer input that uses a 5×5 kernel with 3 input and 16 output channels. These are followed by two convolutional blocks. The first block includes a 5×5 convolutional layer with 16 input and 32 output channels, followed by ReLU activation, batch normalization, and 2×2 max pooling. The second block extends the feature depth to 64 with a 5×5 convolutional layer, again followed by ReLU activation, batch normalization, and max pooling.

The **head pose encoder** processes the head pose images resized to 64×64 pixels. Its architecture begins with a convolutional layer that uses a 5×5 kernel with 3 input and 8 output channels. This is followed by two convolutional blocks. Each block consists of a 5×5 convolutional layer with 8 input and 16 output channels, followed by ReLU activation, batch normalization, and 2×2 max pooling. This design is optimized for extracting the lower-dimensional but essential features from the head pose input.

After feature extraction, tensors from all four encoders are flattened and concatenated into a single feature vector. To do this, the auxiliary scalar feature (estimated distance) is appended, forming a combined input vector of size 45,185. This vector is passed through the fully connected layers. The first fully connected layer maps the input to 256 features, applies the ReLU activation, and is followed by the dropout layer with a probability of 0.2 to prevent overfitting. The next layer reduces the dimensionality to 128 features with another ReLU activation, followed by another dropout layer. Finally, the output layer maps the 128 features to 2 regression targets, representing the model predictions.

The reason we introduce both MultiNet and MultiNet-Base is attributable to the implementation of a more flexible parameter configuration and a reduction in the model overall size suitable for desktop inference. This approach results in a notable reduction in latency, with MultiNet-Base featuring 13,753,298 parameters (52.47 MB model size) in comparison to the MultiNet model, which encompasses 138,440,911 parameters (529.13 MB model size).

#### 3.4.1. Training Details

In this section, we describe the training procedure of MultiNet and MultiNet-Base. We proceed in two phases. First, we freeze the decoder blocks and train the vision encoders, the fusion blocks, and the FC projection head. During this step, we obtain gaze predictions $\hat{g}$ and image reconstructions ${\hat{x}}_{i}$.

Here, we optimize the so-called phase-1 loss that comprises gaze loss and purification loss:

(17) $$\begin{array}{cc} L_{\mathrm{phase}\text{-}1} & =L_{\mathrm{gaze}}+L_{\mathrm{pur}}, \end{array}$$

(18) $$\begin{array}{cc} L_{\mathrm{gaze}} & =∥g-\hat{g}{∥}_{1}, \end{array}$$

(19) $$\begin{array}{cc} L_{\mathrm{pur}} & =1-L_{\mathrm{rec}}. \end{array}$$

In this phase, we make the network learn gaze relevant features first by minimizing $L_{\mathrm{gaze}}$, and secondly, minimizing $L_{\mathrm{pur}}$, whose goal is to make it hard for the decoder to reconstruct image information from the feature map.

Next, we proceed with training the decoders to actually reconstruct the image from the feature map. Here, the aim is simple: optimize $L_{\mathrm{rec}}$ to train decoders to perfectly reconstruct image information (phase-2 loss):

(20) $$L_{\mathrm{phase}\text{-}2}=L_{\mathrm{rec}}={∥x_{i}-{\hat{x}}_{i}∥}_{2}.$$

To make this optimization possible, we freeze the vision encoders to extract feature maps. These feature maps are passed through the decoders that we would like to train.

To sum up, we propose two adversarial phases that optimize the network: one aims to minimize gaze error together with encoders and fusion blocks extracting rich features with gaze essential information, and another for image reconstruction from vision features. We have chosen adversarial-fashion losses that work separately to contribute in better feature extraction for model training. The main idea behind the proposed method is optimal feature extraction due to the multi-branch nature of the method. Lightweight decoders try to reconstruct the input image, deep vision encoders try to extract rich features including gaze information, while the network is optimized to find features that are gaze essential.

#### 3.4.2. Evaluation Metrics

The described gaze estimation methods can return their predictions as either the point of gaze $\hat{p}$ in the screen coordinate system or the gaze direction $\hat{g}$. MSE is a commonly used loss function to quantify the difference between the predicted and actual gaze points and is defined as follows:

(21) $$\mathrm{MSE}=\frac{1}{N}\sum_{i=1}^{N}{\left({\hat{p}}_{i}-p_{i}\right)}^{2}=\frac{1}{N}{∥\hat{p}-p∥}_{2}^{2},$$

where ${\hat{p}}_{i}$, $p_{i}$ is the predicted and corresponding actual gaze point for the *i*-th sample, and *N* is the batch size. By squaring the differences, MSE penalizes larger errors more than smaller ones. This makes it sensitive to outliers and deviations from the true gaze points. To estimate the distance between PoGs in pixels, the root mean square error (RMSE) or Euclidean distance can be used:

(22) $$\mathrm{RMSE}=L_{\mathrm{Euclidian}}=\sqrt{\frac{1}{N}\sum_{i=1}^{N}{\left({\hat{p}}_{i}-p_{i}\right)}^{2}}=\sqrt{\frac{1}{N}{∥\hat{p}-p∥}_{2}^{2}},$$

where ${\hat{p}}_{i}$, $p_{i}$ is the predicted and corresponding actual gaze point for the *i*-th sample, and *N* is the batch size.

Angular error is a standard way to evaluate the difference in gaze direction. Assuming the actual gaze direction is $g_{*}\in R^{3}$ and the estimated gaze direction is ${\hat{g}}_{*}\in R^{3}$, the angular error can be computed as follows:

(23) $$\begin{array}{cc} \cos(\theta ) & =\frac{g_{*}\cdot {\hat{g}}_{*}}{∥g_{*}∥\cdot ∥{\hat{g}}_{*}∥},\\ L_{\mathrm{Angular}} & =\theta =\arccos(\cos(\theta )), \end{array}$$

where ${\hat{g}}_{*}$ and $g_{*}$ are the predicted and corresponding actual gaze directions converted from the original gaze vectors in Euler angles (pitch and yaw) $\hat{g}\in R^{2}$, $g\in R^{2}$, θ is the angle between vectors in degrees, and cos(θ) is the cosine similarity.

### 3.5. Computational Infrastructure

In our experiments, we used two separate infrastructure facilities, i.e., one for model training and validation and another for real-time evaluation of the trained algorithm. The infrastructure for model training is as follows:

- **CPU:** Intel(R) Xeon(R) Gold 6238R CPU @ 2.20 GHz;
- **GPU**: PNY Quadro RTX 6000 with 23,040 MB;
- **RAM**: 251 GB.

The infrastructure for real-time analysis is as follows:

- **CPU**: Intel(R) Core(TM) i5-13400 CPU @ 2.50 GHz;
- **GPU**: Nvidia RTX 4070 with 12,282 MB;
- **RAM**: 16 GB;
- **Web-camera:** Logitech C920 HD Pro, 1080p @ 30 fps–720p @ 30 fps;
- **Depth-camera:** ZED 2i, 2x (1920 × 1080) @ 30 fps.

## 4. Experimental Results

In this section, we compare MultiNet-Base and MultiNet with state-of-the-art methods on the supervised three-dimensional appearance-based gaze estimation task using open-source datasets. Finally, we conduct several experiments to test the effectiveness and stability of the proposed approach, including real-time evaluation and feature visualization.

### 4.1. Comparison with the State-of-the-Art Methods

In order to perform a comprehensive comparison of the developed gaze estimation method, we reproduced the seminal works (see Table 3) in our environment. The number of training epochs is provided below each metric. We focused our attention on the most significant and relevant works in the field, covering not only single input models but multi-branch methods as well. For the gaze estimation datasets, we used MPIIFaceGaze [20], which is an extension of MPIIGaze [49] and Gaze360 [28], as well as our own NLGaze dataset.

**Implementation details.** We employ a deep learning framework (PyTorch Lightning deep learning framework, https://pytorch-lighting.readthedocs.io/en/stable/, PyTorch Lightning 2.5.4 was tested, “URL (accessed on 6 August 2026)”) for experiments and the described preprocessing pipeline with our modifications. We use the Adam optimizer with a learning rate of $10^{-5}$ [52]. The batch size is set to 32 for each model to match the computational limitations of our computing infrastructure. We train MultiNet-base and MultiNet using a Cosine annealing learning rate scheduler [53] and a Step learning rate scheduler for the other methods. The gaze labels used in preprocessing (normalized 3D gaze direction vectors) are converted to 2D gaze direction vectors (yaw, ϕ and pitch, θ):

(24) $$\begin{array}{c} g=\left[g_{x},g_{y},g_{z}\right]\to g=\left(\phi ,\theta \right), \end{array}$$

where $\phi =\arctan_{2}\left(-g_{x},-g_{z}\right)$ and $\theta =\arcsin(-g_{y})$. In this sense, the estimated gaze $\hat{g}\in R^{2}$ is a 2D vector containing the yaw and pitch of the gaze. The images used for all methods were resized to 224×224×3 for fair comparison.

We use L1-loss for the gaze estimation methods considered:

(25) $$L_{gaze}={∥g-\hat{g}∥}_{1}.$$

To compare the results, we implemented a custom data loader for the MPIIFaceGaze and Gaze360 datasets to use with state-of-the-art methods. We added the necessary modalities to each dataset and then compared state-of-the-art methods with our proposed method. We had to add a head pose and face-to-camera distance as described in Algorithm 1. We adopted the leave-one-person-out cross-validation protocol across all participants: the model is trained on one group of subjects, evaluated on another, and tested on a single held-out subject. This protocol is appropriate when the number of available participants is not large enough to support a fixed train/test split [13,27]. We followed this protocol for both MPIIFaceGaze and NLGaze, since NLGaze preserves the same subject-identifier structure as MPIIFaceGaze (p00, p01, …). For Gaze360, we instead used the train/test/validation split provided by the original authors [28].

**Full-Face.** We employed the method described in the original paper [20]. The AlexNet CNN architecture takes the cropped face as input with additional linear regression on top of the last fully connected layer to predict the $\hat{p}$ in the screen coordinates for 2D gaze estimation or normalized gaze vectors $\hat{g}$ for the 3D gaze estimation task.

**GazeTR.** For GazeTR, we used the ResNet-18 convolutional layers for feature extraction. The convolutional layers generate 7×7×512 feature maps from the face images. We then used an additional 1×1 convolutional layer to scale the channel and obtain 7×7×32 feature maps. We fed the feature maps into a six-layer transformer. As for the transformer, we set the hidden size of the two-layer MLP to 512 and performed an eight-headed self-attention mechanism. A 0.1 dropout was used after each MLP.

**PureGaze.** We employed the method described in the original paper [38]. PureGaze contains three networks: a backbone for feature extraction, an MLP for gaze estimation, and an SA-Module for image reconstruction (it contains a block for upsampling and a 1×1 convolution layer for channel alignment). The SA-module is used to reconstruct images and is optimized with reconstruction loss $L_{rec}$ such as the pixel-wise MSE loss. The backbone attempts to prevent the reconstruction. It is optimized with an adversarial loss $L_{adv}=1-L_{rec}$.

The loss functions of the three parts are as follows:

(26) $$\begin{array}{cc} L_{SA} & =L_{rec}={∥I-\hat{I}∥}_{2}, \end{array}$$

(27) $$\begin{array}{cc} L_{MLP} & =L_{gaze}={∥g-\hat{g}∥}_{1}, \end{array}$$

(28) $$\begin{array}{cc} L_{backbone} & =\alpha L_{adv}+\beta L_{gaze}, \end{array}$$

where α=1 and β=3 are hyperparameters, and *I* and $\hat{I}$ are the input cropped and reconstructed face images, respectively.

**EyeNet.** We utilize a simple EyeNet architecture [5] that is basically the ResNet18 vision encoder followed by an MLP applied to each eye patch. The output of each block is extracted, and the mean is taken to estimate the gaze for two eyes.

**iTracker.** Following [10], iTracker processes the cropped face, both eye patches, and a face-grid mask, denoted here as the head pose input $x_{H}$, through four separate branches: weight-sharing convolutional branches for the two eyes, a separate CNN for the face, and fully connected layers for the face-grid mask. The four resulting feature vectors are concatenated and passed through fully connected layers to regress the gaze.

**Dilated-Net.** Following [31], Dilated-Net employs separate VGG16-initialized convolutional backbones for the face and eye branches, with the two eye branches sharing weights. In the eye branches, the deeper pooling stages with dilated convolutions of increasing rate replace the eye branches’ standard pooling to preserve spatial resolution for fine-grained gaze cues, while the face branch retains standard pooling. The features from all three branches are concatenated and regressed through fully connected layers.

**AFF-Net.** We employed the method described in the original paper [51] with a small adaptation: we used the same face-grid mask adopted by iTracker and MultiNet. In the original implementation, the authors of AFF-Net designed a geometric branch that uses three independently tracked bounding boxes, one each for the face, left eye, and right eye. These are passed through a fully connected layer to obtain geometric features, which are concatenated with the face-encoder output to form a vector that adaptively modulates the eye-branch feature maps via adaptive group normalization (AGN), conditioning the eye representation on where the face and eyes are located in the image. In our adaptation, we instead rely on a single face-grid mask. To preserve AFF-Net’s geometric-conditioning pathway without altering its core fusion mechanism, we recover one bounding box from this mask, based on its spatial extent, and use it in place of the three original per-region boxes. Rather than fusing modalities in a single step at the end, AFF-Net conditions the eye branches on face and geometry features through AGN twice, once per eye and again when merging the two eyes. The three resulting feature streams are then concatenated and passed through the final regression layer. Despite sharing the AFF acronym, this mechanism sits at a different stage of the pipeline and follows a different formulation than the fusion adopted in MultiNet (Section 3.4).

**MultiNet-Base and MultiNet.** We use both models with all artificial modalities, including cropped face, left eye, right eye, estimated distance, and head pose.

**Note on the Architectural Innovations.** The MultiNet and MultiNet-Base models draw heavily on prior multi-branch methods: iTracker, Dilated-Net, and AFF-Net [10,31,51]. Three points distinguish MultiNet and MultiNet-Base from them.

First, input modality: none of the three baselines explicitly encodes a metric quantity, whereas both MultiNet and MultiNet-Base take the estimated face-to-camera distance as an additional input, encoded as a pseudo-depth map $x_{D}$. Second, the feature extraction backbone: iTracker, Dilated-Net, and AFF-Net all use comparatively shallow, weight-shared towers (AlexNet-derived, VGG16-initialized, and a bespoke GroupNorm/Squeeze-and-Excitation design, respectively); MultiNet-Base retains this same lightweight philosophy but drops weight sharing between the two eyes, while MultiNet substitutes five independently parameterized ResNet50 encoders paired with reconstruction decoders used only during training, giving it substantially more representational capacity. Third, and most critical, is the fusion strategy: iTracker and Dilated-Net flatten and concatenate every stream’s features with no learned cross-modal weighting; MultiNet-Base deliberately retains this same concatenation-based fusion, so that its comparison against MultiNet in the comparative study (see Table 3) isolates the contribution of attentional fusion from naive concatenation. AFF-Net’s AGN conditions only the eye branch, and the final combination of eye, face, and geometry features is still a plain concatenation. MultiNet, in contrast, applies the attentional feature fusion (AFF) block of [48] symmetrically across all five modalities, gating every pairwise merge with a learned, sample-dependent weight, and is further trained with a two-phase adversarial procedure (Section 4) that purifies gaze-irrelevant information, a step for which iTracker, Dilated-Net, and AFF-Net offer no equivalent.

**Comparative Study.** Table 3 shows a comparative study with state-of-the-art methods. MultiNet achieves state-of-the-art performance on two of the presented datasets (Gaze360 and NLGaze), with an angular error of 7.47° and 7.58°, respectively, while the MultiNet-Base method is comparable to state-of-the-art performance. The performance of MultiNet on NLGaze is evidence of model generalization on a dataset with a natural environment. The improved result on Gaze360 means that our method is robust to samples with varying head pose, due to the specificity of the data. Moreover, the quality of image samples in Gaze360 is significantly less detailed and much noisier than in NLGaze or MPIIFaceGaze. On the other hand, our method is not the best on MPIIFaceGaze (but close), while the best result is achieved by the simplest of the presented methods, EyeNet. EyeNet is a model that consists of two ResNet18 feature extractors for each image input (pair of eyes) followed by an MLP. This behavior can be explained by considering that data in MPIIFaceGaze is considerably heterogeneous. Data were collected using custom software running as a background service on the participants’ laptop devices with different cameras for each user presented in the dataset.

Among the three multi-branch baselines, Dilated-Net comes closest to MultiNet on NLGaze (7.95° vs. 7.58°), while iTracker and AFF-Net trail further behind at 8.59° and 8.79°, respectively. On MPIIFaceGaze, AFF-Net is in fact the strongest baseline overall (2.62°), narrowly surpassing MultiNet (2.94°) and performing comparably to iTracker (2.99°) and Dilated-Net (3.15°). On Gaze360, AFF-Net ties with PureGaze for second place (8.01°), still trailing MultiNet’s 7.47°. MultiNet is therefore the best or third-best method on every dataset considered, and this advantage cannot be attributed merely to a larger set of input modalities, given that iTracker and AFF-Net receive the same cropped face, eyes, and head pose inputs.

The angular error of MultiNet-Base on the NLGaze dataset is substantially higher than that of every other method in the comparison. We investigated this discrepancy further. iTracker, the architecturally closest baseline, is also multi-branch and fused with plain concatenation, yet it reaches 8.59° on NLGaze; concatenation-based fusion alone cannot explain MultiNet-Base’s 14.02°. It is tempting to instead blame the distance modality, since that is what iTracker lacks; however, our own ablation study (Table 4) rules this out on its own: holding face, eyes, and head pose fixed under concatenation, adding distance improves the NLGaze result rather than harming it (12.51° to 10.33°, rows 9 and 11). A shallow, non-pretrained backbone is not the explanation in isolation either, since Dilated-Net and AFF-Net also use shallow, non-pretrained encoders with concatenation-based (or partially adaptive) fusion and show no comparable failure. What is unique to MultiNet-Base, and not exercised anywhere else in this comparison, is the combination of all three factors at once: a shallow, non-pretrained backbone reconciling five heterogeneous streams, including the estimated distance quantity appended through unweighted concatenation. Every other configuration in Table 3 and Table 4 relaxes at least one of these three factors: the shallow baselines omit the distance stream, and the ablation study’s concatenation-based configuration pairs distance with MultiNet’s stronger ResNet50 backbones; none of them fails this way. We do not have a controlled experiment isolating this specific combination and flag it as a direction for future research.

### 4.2. Ablation Study

Table 4, shows an ablation study on MultiNet to analyze the contribution of different input modalities to gaze estimation performance. There are three groups of tests in this table, numbered 1–4, 5–8, and 9–12. We aim to check the following in each test group:

1. Whether the method with AFF blocks works better than simple concatenation for modality fusion;
2. Whether adding the distance to input modalities improves gaze estimation performance.

For each test, the table shows the best result (angular error) in bold, giving us the best configuration of method and input modalities. Let us consider each group one by one. In tests 1–4, we use the eyes pair as the main modality. If we compare 1 to 2 and 3 to 4, we observe that using the AFF blocks for fusion achieves, on average, a lower angular error (in two out of the three datasets) than simple concatenation. Similarly, by comparing 1 to 3 and 2 to 4, we observe that adding distance estimation generally improves performance for both concatenation and fusion.

In test groups 5–8, the main modalities are images of cropped faces and eyes. Following the same procedure described above, we again observe that the fusion blocks perform better on average (tests 6 and 8) than concatenation (tests 5 and 7). On the other hand, the addition of distance estimation positively improves the performance of test 8 in comparison to test 6, while for tests 5 and 7, adding distance improves the metrics only on MPIIFaceGaze.

Finally, test groups 9–12 use all modalities. The results here are more consistent, with the fusion blocks outperforming concatenation and distance estimation improving performance across all datasets. Overall, we can conclude that the MultiNet configuration with attentional feature fusion (AFF) blocks works better than the version with concatenation of flattened features. Moreover, adding distance to input modalities generally improves gaze estimation performance.

We have noticed that adding the estimated metric distance does not consistently improve performance. Of the six base/plus-distance comparisons in Table 4, three are inconsistent: tests 2 to 4 worsen Gaze360 (7.74° to 8.07°); tests 5 to 7 worsen both Gaze360 (7.55° to 8.42°) and NLGaze (11.29° to 14.98°); tests 6 to 8 worsen MPIIFaceGaze (3.39° to 4.26°). The remaining three are fully consistent, improving performance on all three datasets: tests 1 to 3, and both comparisons that already include head pose, tests 9 to 11 and tests 10 to 12.

Every inconsistent comparison lacks head pose and additionally involves either the face branch (tests 5–7 and 6–8) or attentional gating (tests 2–4); the only comparison without head pose that is nonetheless fully reliable is the simplest one, plain concatenation of eyes and distance alone (tests 1–3). Once head pose is included as an input, adding distance is uniformly beneficial across every dataset and both fusion mechanisms (test groups 9–12). We read this as evidence that distance and head pose act as complementary geometric cues rather than independent ones: a given face-to-camera distance corresponds to a different real-world spatial configuration depending on head orientation, so distance is an ambiguous signal unless head pose provides the context for it.

### 4.3. Feature Visualization

**Figure 6:**
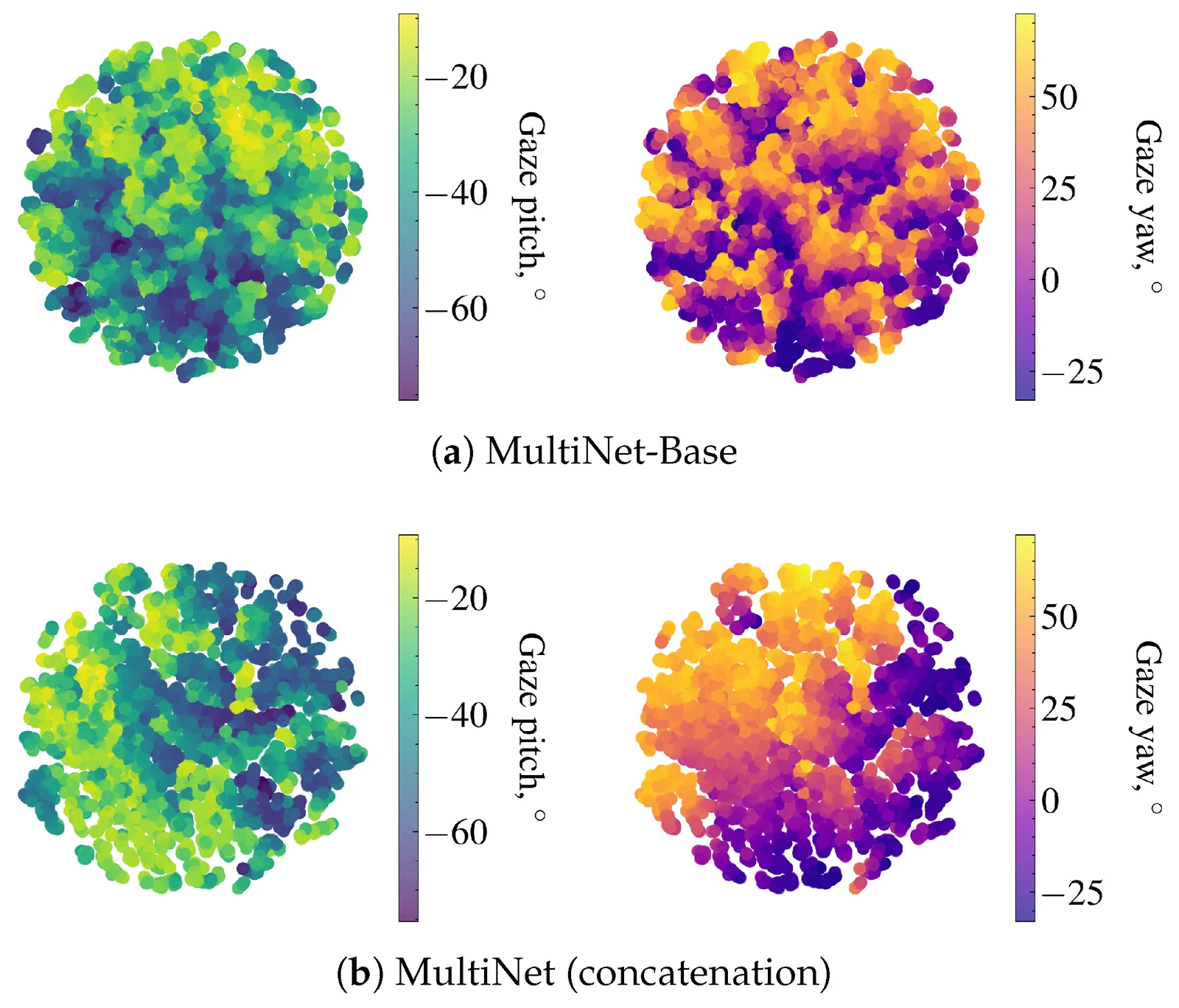

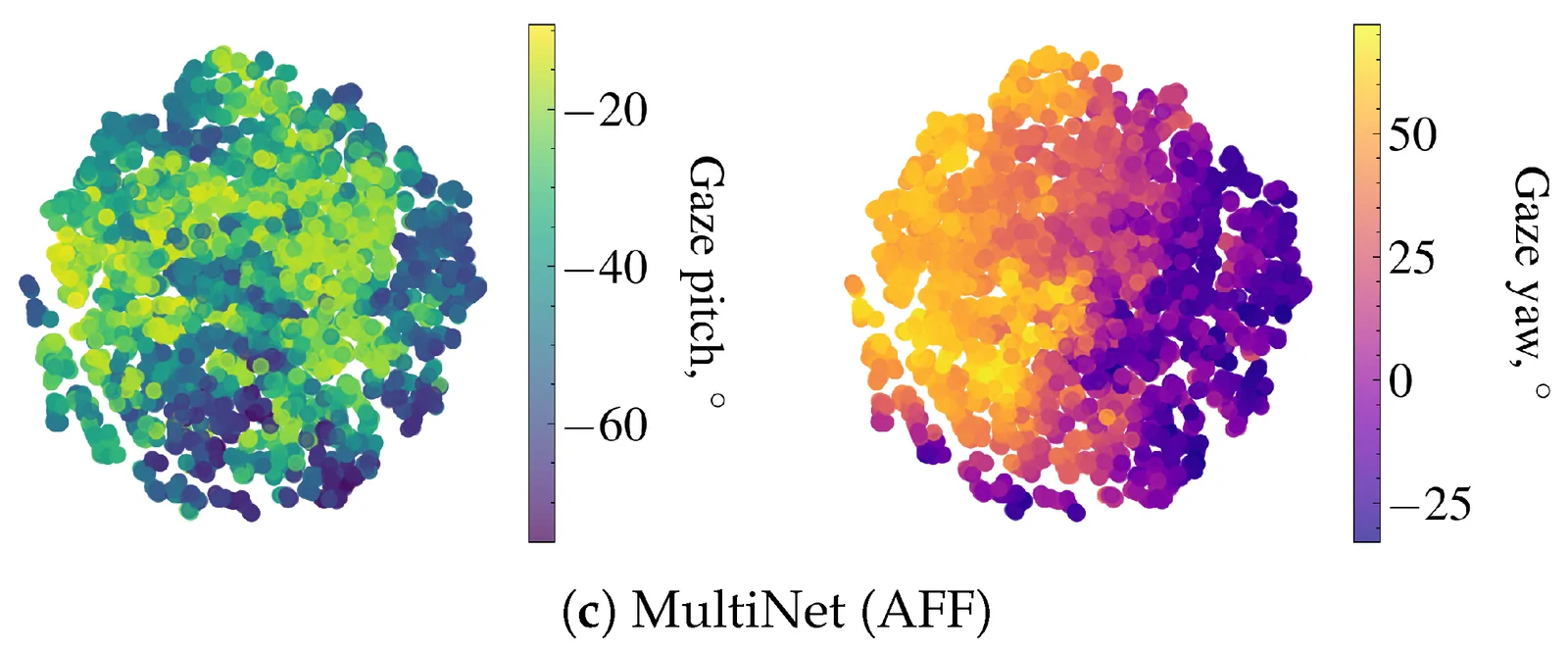
Illustration of the features on MultiNet-Base and MultiNet, where different colors indicate different true gaze pitch and yaw.

To understand the effectiveness of developing a good representation in an intuitive way, we visualize the distribution of features after fusion on the gaze estimation task on MPIIFaceGaze with t-SNE [54]. The generated features from three of our different models are presented in Figure 6. We visualize pitch and yaw gaze angles in color, where feature points with close gaze direction share a similar color. Despite having some level of color gradation for pitch, MultiNet-Base (Figure 6a) does not perform as well for yaw, where purple areas abruptly appear in orange areas. In contrast, MultiNet with concatenation (Figure 6b) and AFF blocks for feature fusion (Figure 6c) learn better features, as shown by smoother color gradation in pitch and yaw gaze directions.

### 4.4. Gaze Estimation Stability

In this section, we demonstrate the stability of the proposed method by showing the influence of adding noise to the input images, to the input distance, and to the landmarks for distance estimation.

**Figure 7:**
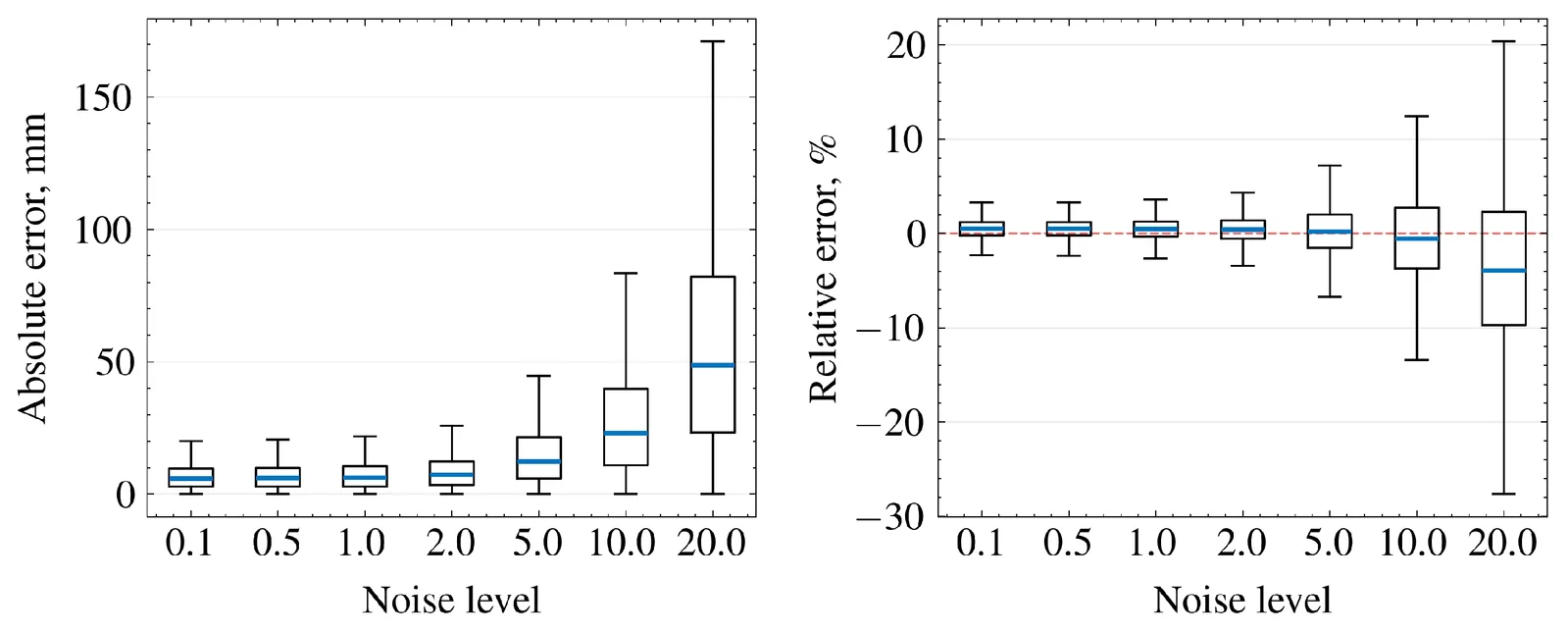
Dependence of absolute (**left**) and relative (**right**) angular error from the level of Gaussian noise applied to facial landmarks (which directly affect distance estimation).

**Applying Noise to Facial Landmarks.** To quantify the sensitivity of the distance estimator, whose estimations directly rely on facial landmarks, we apply Gaussian noise to facial landmarks with different levels of noise. Figure 7 shows the degradation of the estimated distance with increasing levels of Gaussian noise (thus resulting in deviations in distance estimation).

**Figure 8:**
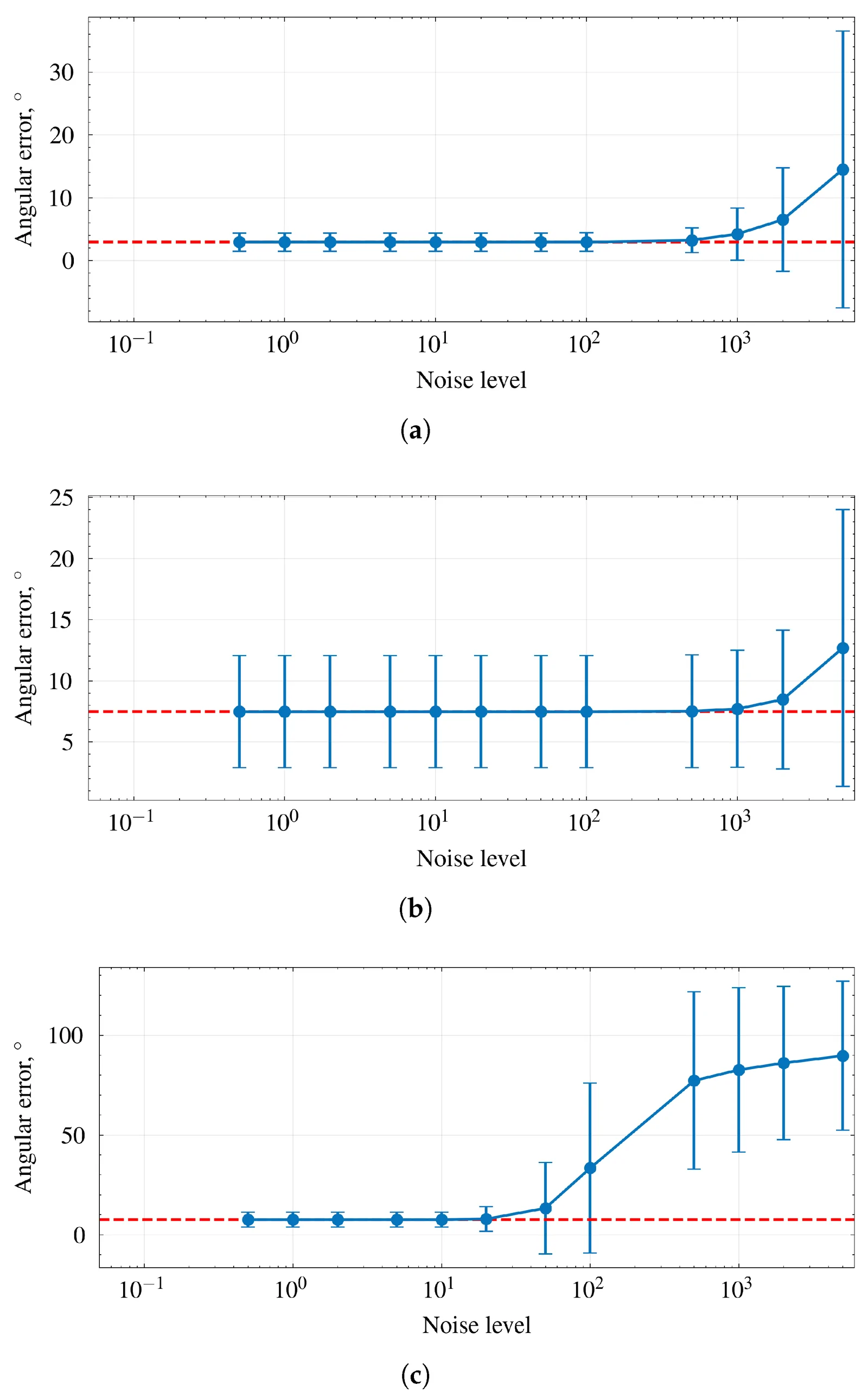
Stability analysis of adding Gaussian noise to distance input in MultiNet. (**a**) Dependence of angular error from noise applied to input distance on MPIIFaceGaze; (**b**) dependence of angular error from noise applied to input distance on Gaze360; (**c**) dependence of angular error from noise applied to input distance on NLGaze.

**Applying Noise to Distance.** Further, we demonstrate a sensitivity analysis for the proposed method with noisy distance inputs in Figure 8a–c.

**Figure 9:**
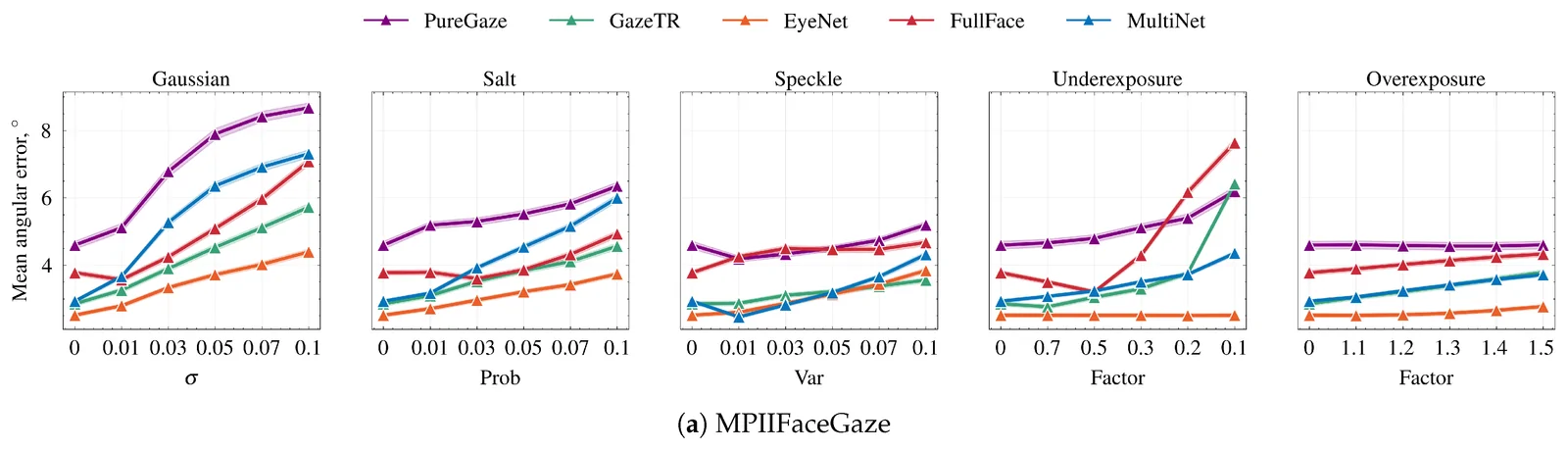

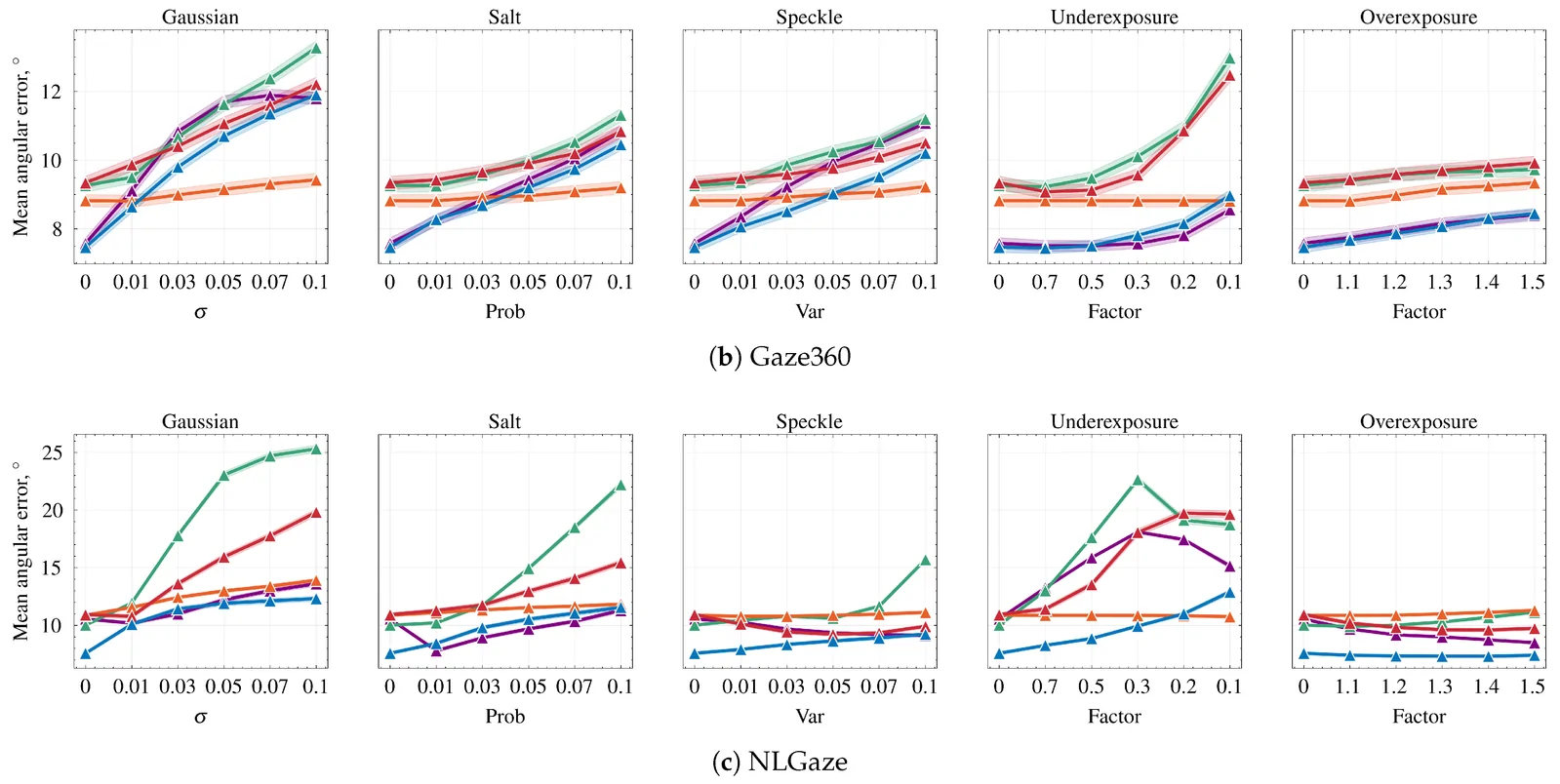
Gaze estimation errors under different types of noise applied to input image on MPIIFaceGaze, Gaze360, and NLGaze. The content inside graphs represents the variation in the mean angular error with corresponding noise. Baseline (stated as 0 for each experiment with noise) represents the estimation error without noise.

**Figure 10:**
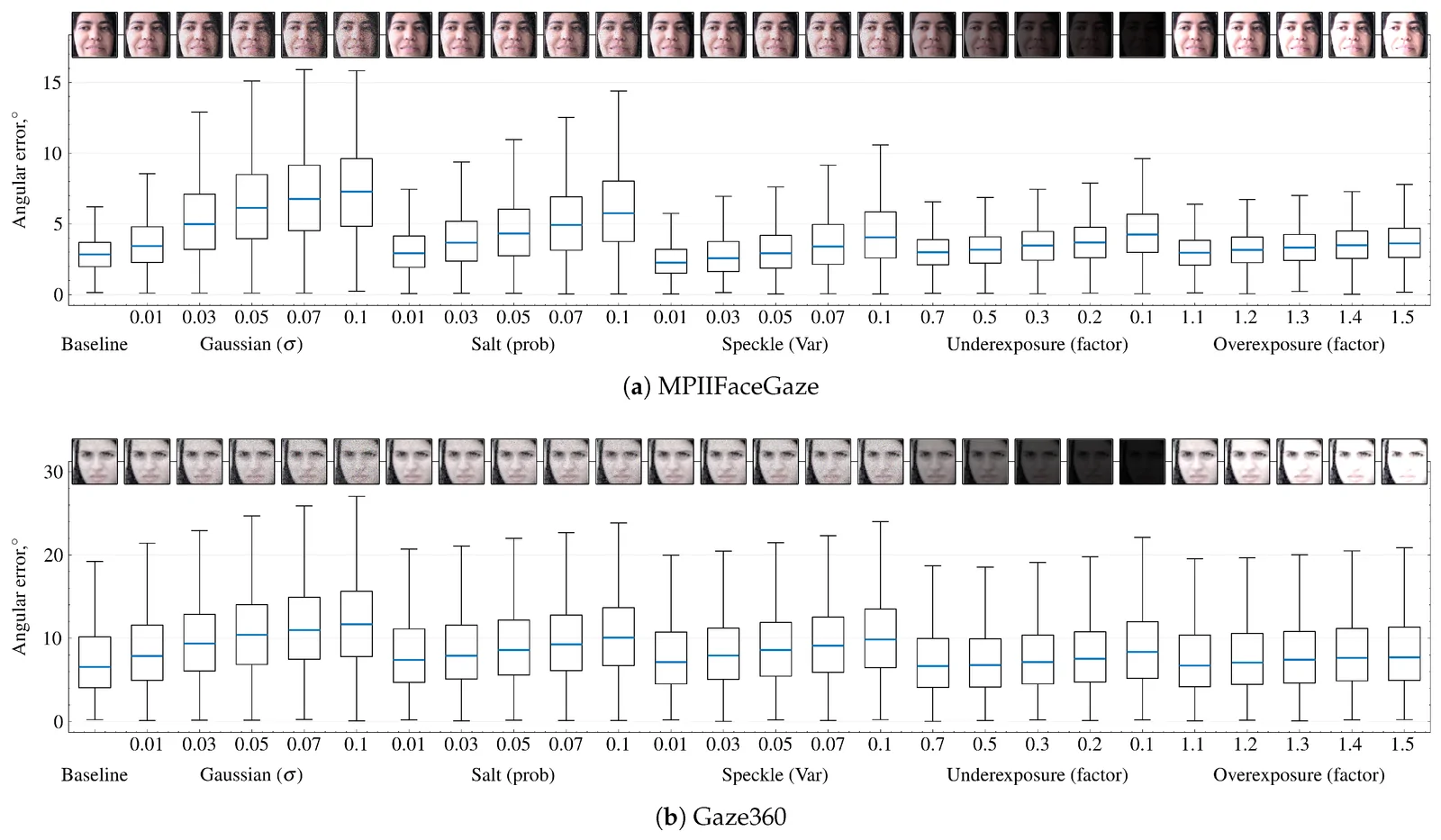

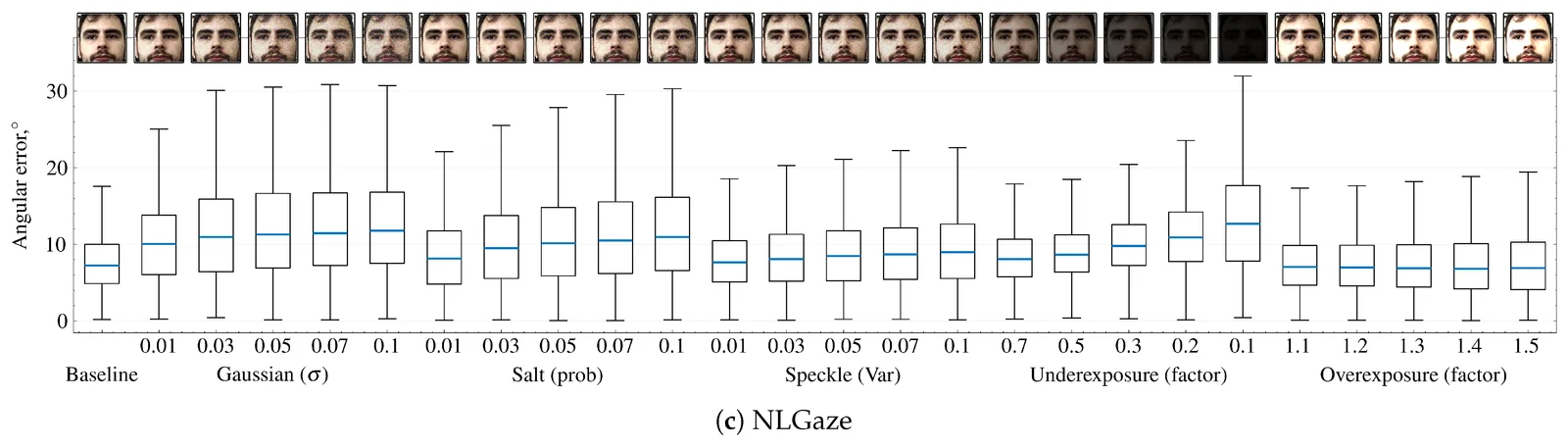
Gaze estimation errors under different types of noise applied to input image on MPIIFaceGaze, Gaze360, and NLGaze. The content inside graphs represents the variation in the angular error with corresponding noise. Images on the top of each graph represent the original and corrupted samples. Baseline represents the estimation error without noise.

**Applying Noise to Input Images.** To quantify the method stability, we apply different amounts of noise to the input image for MPIIFaceGaze, Gaze360, and NLGaze and test the PureGaze, GazeTR, EyeNet, FullFace, and MultiNet models. As shown in Figure 9a–c, MultiNet demonstrates comparable results against different types of noise. For better reference, a more detailed version of the same experiment for MultiNet only is shown in Figure 10a–c.

In general, we observe that MultiNet trained on NLGaze is less sensitive to noise, while it performs as well as or, in many cases, better than other methods on the other datasets. In particular, MultiNet is generally rather stable to changes in illumination (MultiNet is in the top three methods across all datasets in under- and over-exposure tests (Figure 9a–c); however, model performance is more affected by under-exposed rather than over-exposed inputs). For facial landmarks (Figure 7) and distance (Figure 8a–c), we see that the method is again stable until a threshold level of noise is reached that significantly degrades performance. These results indicate a certain degree of robustness against adverse data alteration.

### 4.5. Real-Time Evaluation

Real-time evaluation is crucial for understanding the accuracy of the trained model in real-life scenarios. For this purpose, the full-HD display was divided into rectangular areas for the assessment of gaze estimation. The division of the screen into areas with stimuli is inspired by calibration patterns, a standard way to calibrate eye-tracking systems [55,56]. This division was empirically determined, and a 6×5 grid (five columns and six rows) for screen splitting was deemed sufficient.

The objective during real-time evaluation is to observe a colorful stimulus in the form of a cross to record the model predictions. The user is positioned approximately 80 cm from the screen during the evaluation and instructed to continuously focus on the colorful cross. The cross changes color based on precise timings. Initially, the cross appears in a random rectangular area in red, with no gaze fixation acquisition. After 3 s, it turns green, meaning that the program registers fixations. Recording proceeds for 2 s, then the cross changes color to orange for 1 s, meaning that a new cross position will appear soon. This procedure repeats until all fixation rectangle areas are covered. An error of 50 pixels in such a setting corresponds to approximately 15.7 mm.

**Figure 11:**
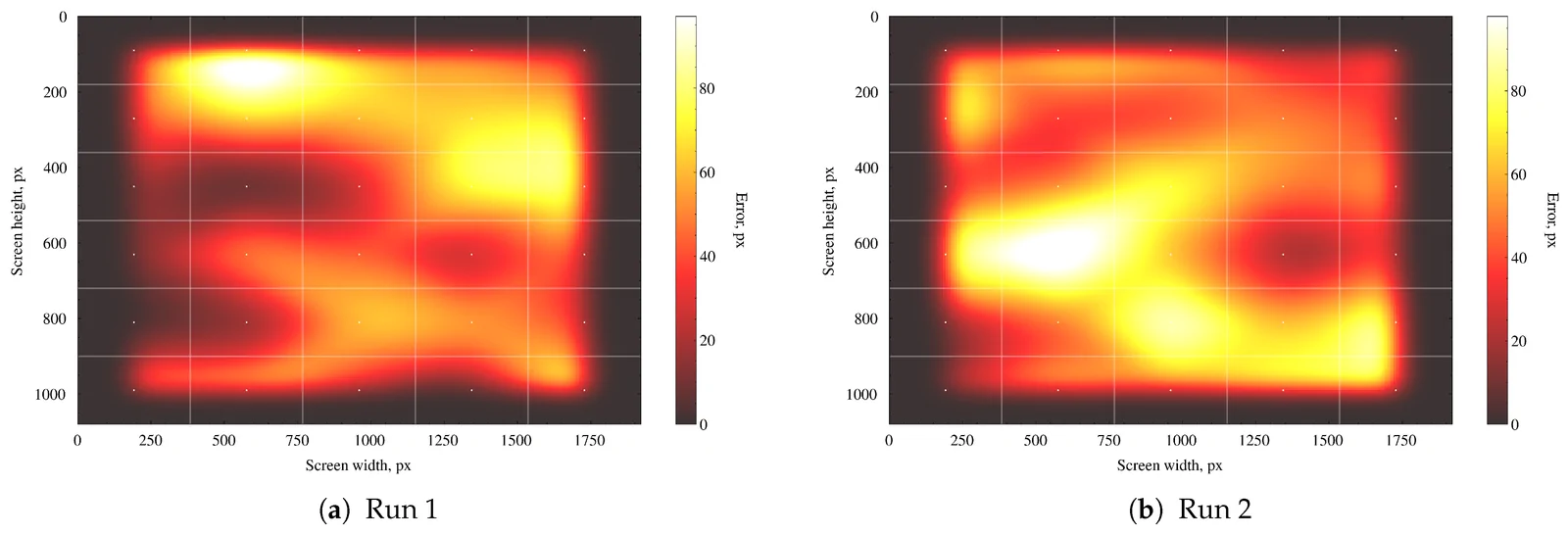
Inference showcase of MultiNet-Base trained on NLGaze using PoG error interpolated heatmaps, 6×5 grid.

We provide point-of-gaze heatmaps as the main visual evaluation tool, which were obtained by interpolating point-of-gaze grid errors (Figure 11). It can be observed from Figure 11 that the model achieves an RMSE lower than 100 pixels, resulting in high-accuracy predictions. In particular, it achieves an average RMSE of 43.01 px (∼13.48 mm) for the run on Figure 11a and RMSE of 43.22 px (∼13.56 mm) for a second run on Figure 11b. Nevertheless, the heatmaps are not entirely consistent, pointing out some problem areas around the fixations closer to the corners of the screen.

Additionally, a moving average filtering technique is implemented to refine the predictions. This involves continuously adding model predictions to a fixed queue and calculating the average of the queue each time the prediction is visualized. By smoothing fluctuations in the predictions over time, this approach enhances the stability and reliability of the gaze estimation system, improving the overall user experience and accuracy of gaze tracking.

Finally, the Student’s *t*-test was performed to assess the statistical significance of the discrepancy between the model predictions and the reference stimulus, yielding p<0.05 for Figure 11a,b. This indicates that the difference between the predictions and the reference points is statistically significant for both cases.

### 4.6. Real-Time Pipeline Latency

**Figure 12:**
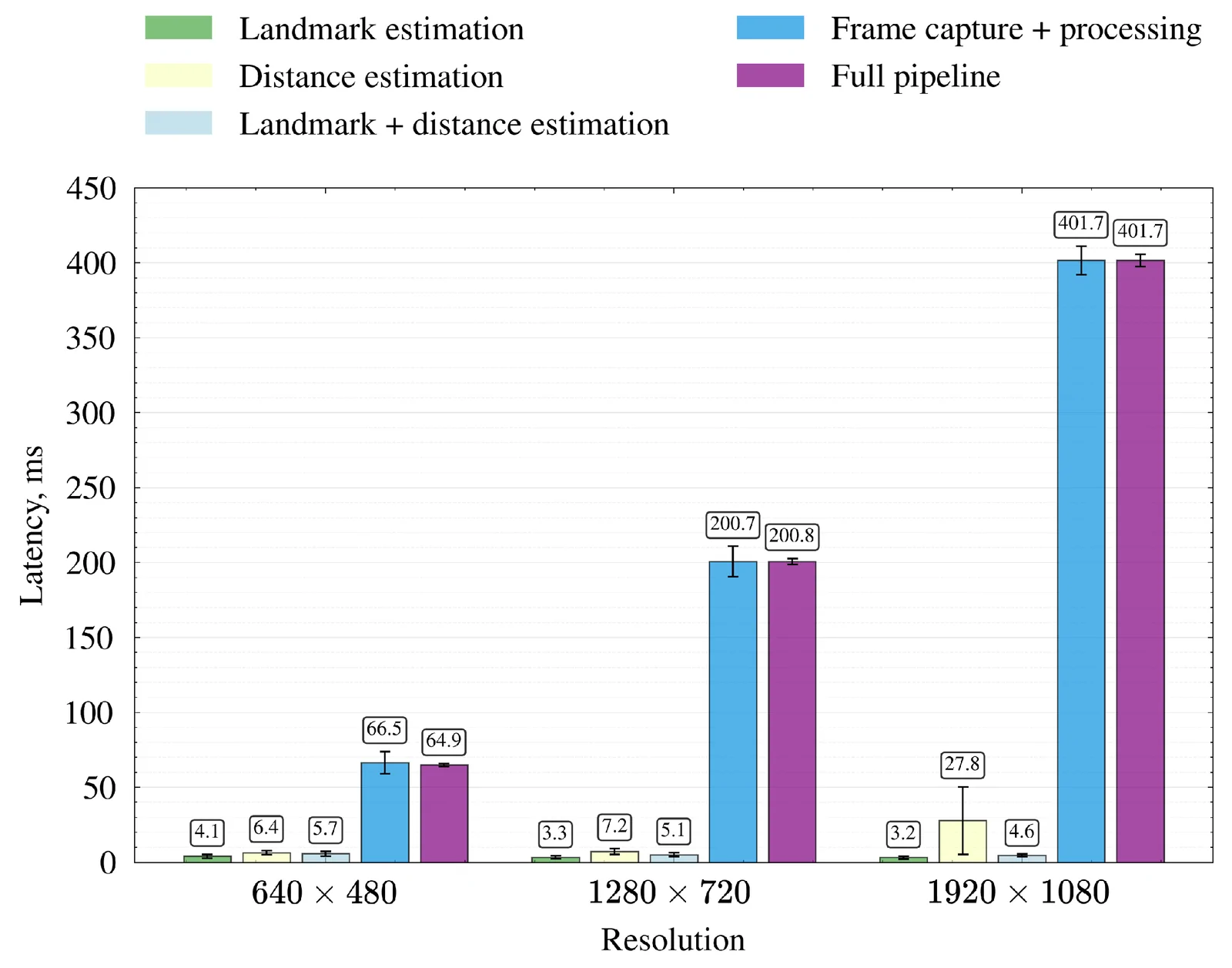
Pipeline latency.

Understanding the latency of each block of the pipeline is crucial for reproducibility and further optimization. In this section, we present simple yet valuable information regarding the latency of each block of the presented real-time pipeline. We divide the full pipeline into four components: frame capture and preprocessing, face landmark estimation, distance estimation, and the deep learning model. Figure 12 shows the results for different image resolutions. The latency of the deep learning model is 1.45±0.39 ms (FPS 724.84±130.51). We do not show this in Figure 12 because its performance is independent of the input image resolution, since it takes already preprocessed images of smaller size.

It can be seen in Figure 12 that real-time performance is not affected by the facial landmark extraction or distance estimation algorithms. Instead, the bottleneck is the frame capturing and processing block (66.5 ms or 15 FPS). For this reason, we use 640×480 resolution for video capturing, as only in such settings can we obtain a decent FPS applicable for real-time. This is probably connected with the way we extract the frames from the video stream and the video capture itself (the maximum framerate that the camera can achieve according to the datasheet is 30 FPS).

We point out that the blocks of the processing pipelines are implemented on multiple threads that run in parallel, which explains why the overall performance is not equal to the sum of the latencies.

## 5. Discussion

Across the three datasets considered, MultiNet is consistently the best- or third-best-performing method, and this advantage does not simply follow from access to more input modalities: AFF-Net and iTracker receive the same cropped face, eyes, and head pose inputs, yet neither matches MultiNet’s overall performance. This points to the attentional feature fusion mechanism and the two-phase adversarial training procedure as the source of the improvement, rather than the mere availability of additional input signal.

The ablation study complicates this picture in an informative way. When head pose is present, adding distance helps consistently across every dataset and both fusion strategies; when it is absent, the two geometric cues interact unpredictably, sometimes helping and sometimes hurting performance. Face-to-camera distance therefore does not appear to be an independent cue, but rather one that becomes informative only once head orientation places it in context, so distance and head pose are best understood as complementary rather than additive.

## 6. Limitations and Future Work

The proposed distance estimation approach assumes a calibrated camera and a face visible within the full frame; it is therefore not directly applicable to datasets that provide only pre-cropped face images, which limits its use as a drop-in addition to existing appearance-based pipelines.

We assume that, in the current form, the method is applicable to environments without under- and over-exposed illumination conditions. As far as metric distance estimation is based on a facial landmark estimation algorithm, it is known to be sensitive to variations in lighting. Thus, the proposed method is applicable in natural environments with sufficient illumination or/and the imaging system with a high dynamic range.

The real-time evaluation of MultiNet-Base also exposes two practical boundaries. Point-of-gaze predictions are visibly less reliable near the corners of the screen than toward its center, likely reflecting the lower density of extreme gaze angles in the training data. In addition, frame capture remains the bottleneck of the real-time pipeline, which caps the usable resolution at 640×480 pixels; higher-resolution input, although shown to improve accuracy, is not currently compatible with real-time operation on our hardware.

The evaluation protocol further assumes a broadly static user position, approximately 80 cm from the screen and facing the camera; performance under substantial head movement, extreme poses, or occlusion of the eyes or face has not been characterized and remains an open question. Finally, MultiNet’s accuracy comes at a computational cost, since its 138M-parameter backbone is more than an order of magnitude larger than MultiNet-Base, which constrains its suitability for resource-constrained or embedded deployment.

Furthermore, the data were gathered in natural environments, specifically laboratory rooms equipped with window shades and ample natural light streaming in from outside. The collected dataset has a natural bias towards working hours, not covering challenging illumination presented with over- or under-exposed samples. The illumination levels were not controlled explicitly and were not measured.

In the scope of future work, we would like to continue to dive into the depth-aware methods and finding another ways of feature fusion without explicit multi-branch methods. This reasoning is supported by the empirical findings that increasing the number of branches often leads to higher computing requirements and require significant effort in adapting accurate methods to the real-time performance required for online gaze estimation.

## 7. Conclusions

In this article, we have proposed a gaze estimation approach that provides a generalizable solution for gaze estimation using metric distance estimation as conditioning.

The proposed method is based on two networks: MultiNet and MultiNet-Base. MultiNet is a CNN-based architecture with attentional feature fusion for multiple input modalities and an adversarial training procedure for valuable feature extraction for gaze estimation. Conversely, MultiNet-Base is a lightweight version of MultiNet with concatenation of flattened feature vectors with standalone model optimization.

The presented MutiNet CNN model was trained on the NLGaze dataset with attentional feature fusion from multiple input images (cropped face, left and right eyes, and head pose frames) and estimated distance, which demonstrated the best performance. The proposed method was compared with state-of-the-art solutions including Full-Face, EyeNet, GazeTR, iTracker, Dilated-Net, AFF-Net, and PureGaze on MPIIFaceGaze, Gaze360, and our proposed datasets. Our method demonstrated state-of-the-art performance on the Gaze360 and NLGaze datasets, achieving an angular error of 2.94°, 7.47°, and 7.58° on MPIIFaceGaze. At the same time, experiment results showed that the proposed MultiNet solution is noise resistant.

The initial objective was to accurately project the point-of-gaze predictions in close proximity to the reference points displayed on the screen. Nevertheless, the calibration-free method demonstrated that meeting the specified task requirements was feasible within restricted user behavior.

In terms of the ablation study, we demonstrated that the attentional feature fusion mechanism and the addition of estimated distance to the set of input modalities result in significant angular error reduction. We assumed that, with the recent advancements in zero-shot relative and metric depth estimation works, the method can be further improved. This can be done by using the distance map from the full frame image instead of the depth map approximation using a scalar, as proposed in this work.

The proposed approach can be deployed on off-the-shelf web cameras and used in real time and in natural environments for gaze estimation. Its reliable use nevertheless requires visible facial landmarks, moderate head motion, and sufficiently stable illumination; severe occlusion, reflections, extreme poses, or poor calibration can reduce accuracy. The proposed multi-branch learning method, together with the estimated metric distance from the input image, is a bridge that paves the way to gaze estimation in unconstrained, natural settings, with applicability to a single RGB camera in contrast to previous works that use RGBD cameras. 

## Figures and Tables

**Table 1:** Summary of gaze estimation datasets.

Dataset	Visual Content	Total	2D Gaze	3D Gaze	NIR
MPIIGaze [13]	images	213 K	-	+	-
MPIIFaceGaze [20]	images	45 K	+	+	-
Gaze360 [28]	images	172 K	-	+	-
ETH-XGaze [14]	images	1.1 M	+	+	-
NVGaze [43]	images	4.5 M	-	+	+
RT-GENE [44]	images	123 K	-	+	-
GazeCapture [10]	images	2.4 M	+	+	-
EVE [5]	videos	4.2 K	+	+	-
NLGaze **(Our study)**	images	141 K	+	+	-

**Table 2:** Notation for the proposed image normalization.

Notation	Description
$d_{orig}$	face-to-camera distance after solving the perspective-n-point (PnP) problem (as outlined in Algorithm 1)
$d_{n}$	distance that is desired and fixed for eye patch normalization (700 mm in this case)
θ	head rotation angle, defined between global parallel line and line connecting the eyes centers landmarks
$x_{l},y_{l}$	left eye center coordinate
$x_{r},y_{r}$	right eye center coordinate
*h*	desired output shape of the normalized patch, which in this case is 64 to accommodate the eye and/or face images, which are represented by $x_{L}\in R^{3\times 64\times 64}$, $x_{R}\in R^{3\times 64\times 64}$ and/or $x_{F}\in R^{3\times 64\times 64}$
$t_{x},t_{y}$	translation offset to ensure fixed position of eyes on cropped eye and/or face images
$d_{des}$	desired fixed distance for normalized face patch ($d_{des}=0.5\cdot h$)
$d_{eyes}$	distance between the centers of the eyes, calculated using the Euclidean distance formula: $d_{eyes}={∥\left(x_{l},y_{l}\right)-\left(x_{r},y_{r}\right)∥}_{2}$

**Table 3:** Comparative study with state-of-the-art methods, angular error on test (**1st** and 2nd best results are shown for each dataset).

Method	Input Modalities	MPIIFaceGaze [20]	Gaze360 [28]	NLGaze
Full-Face [20]	Cropped face	3.78° (6 epochs)	9.35° (9 epochs)	10.94° (7 epochs)
EyeNet * [38]	Eyes	**2.52°** (12 epochs)	8.82° (12 epochs)	10.92° (9 epochs)
GazeTR [50]	Cropped face	2.86° (8 epochs)	9.27° (12 epochs)	10.07° (9 epochs)
PureGaze [38]	Cropped face	4.60° (7 epochs)	8.01° (19 epochs)	10.55° (15 epochs)
iTracker [10]	Cropped face, eyes, head pose	2.99° (9 epochs)	8.47° (13 epochs)	8.59° (10 epochs)
Dilated-Net [31]	Cropped face, eyes	3.15° (17 epochs)	8.24° (14 epochs)	7.95° (19 epochs)
AFF-Net [51]	Cropped face, eyes, head pose	2.62° (5 epochs)	8.01° (7 epochs)	8.79° (10 epochs)
MultiNet-Base **(Our study)**	Cropped face, eyes, head pose, distance	3.07° (5 epochs)	9.37° (8 epochs)	14.02° (11 epochs)
MultiNet **(Our study)**	Cropped face, eyes, head pose, distance	2.94° (17 epochs)	**7.47°** (20 epochs)	**7.58°** (10 epochs)

* Angular error on EyeNet is a mean from angular error for each eye.

**Table 4:** Ablation study on MultiNet, angular error on test.

№	Method	Input Modalities	Numberof AFF Blocks	MPIIFaceGaze [20]	Gaze360 [28]	NLGaze
1	MultiNet (concatenation)	Eyes	1	4.74° (7 epochs)	8.10° (19 epochs)	**13.08°** (3 epochs)
2	MultiNet (AFF)	Eyes	1	**3.84°** (12 epochs)	**7.74°** (18 epochs)	13.13° (4 epochs)
3	MultiNet (concatenation)	Eyes, distance	2	4.34° (8 epochs)	**7.99°** (19 epochs)	11.26° (12 epochs)
4	MultiNet (AFF)	Eyes, distance	2	**3.36°** (10 epochs)	8.07° (15 epochs)	**8.69°** (3 epochs)
5	MultiNet (concatenation)	Cropped face, eyes	2	4.92° (12 epochs)	**7.55°** (15 epochs)	11.29° (11 epochs)
6	MultiNet (AFF)	Cropped face, eyes	2	**3.39°** (16 epochs)	7.61° (19 epochs)	**10.13°** (11 epochs)
7	MultiNet (concatenation)	Cropped face, eyes, distance	3	**4.09°** (10 epochs)	8.42° (14 epochs)	14.98° (4 epochs)
8	MultiNet (AFF)	Cropped face, eyes, distance	3	4.26° (17 epochs)	**7.56°** (19 epochs)	**9.16°** (12 epochs)
9	MultiNet (concatenation)	Cropped face, eyes, head pose	3	3.88° (12 epochs)	7.86° (19 epochs)	12.51° (13 epochs)
10	MultiNet (AFF)	Cropped face, eyes, head pose	3	**3.53°** (9 epochs)	**7.66°** (18 epochs)	**11.35°** (9 epochs)
11	MultiNet (concatenation)	Cropped face, eyes, head pose, distance	4	3.41° (17 epochs)	7.79° (10 epochs)	10.33° (15 epochs)
12	MultiNet (AFF)	Cropped face, eyes, head pose, distance	4	**2.94°** (17 epochs)	**7.47°** (20 epochs)	**7.58°** (10 epochs)

Bold font is used for displaying the best result on the specific dataset for the pair of tests indicated with **№** (1-2, 2-3, etc.), e.g., in 1-2 on eyes as input modalities, the best result is MultiNet (AFF) on MPIIFaceGaze, the same on Gaze360, and MultiNet (concatenation) on NLGaze.

## Data Availability

The original contributions presented in this study are included in the article. Further inquiries can be directed to the corresponding author.

## Abbreviations

The following abbreviations are used in this manuscript:

CNN	Convolutional neural network
MSE	Mean squared error
RMSE	Root mean squared error
FPS	Frames per second
RGB	Red, green, blue
RGBD	Red, green, blue + depth, abbreviation of depth camera
PoG	Point of gaze
MAPE	Mean absolute percentage error
PnP	Perspective-n-point problem
AFF	Attentional feature fusion
MS-CAM	Multi-scale channel attention module
MLP	Multilayer perceptron
FC	Fully connected layer
